# Joining soft tissues to bone: Insights from modeling and simulations

**DOI:** 10.1016/j.bonr.2020.100742

**Published:** 2020-12-23

**Authors:** Alexandra Tits, Davide Ruffoni

**Affiliations:** Mechanics of Biological and Bioinspired Materials Laboratory, Department of Aerospace and Mechanical Engineering, University of Liège, Liège, Belgium

**Keywords:** Enthesis, Bone-tendon interface, Modeling, Computer simulations, Fibrocartilage, Gradients, Bimaterial attachment, Finite element analysis

## Abstract

Entheses are complex multi-tissue regions of the musculoskeletal system serving the challenging task of connecting highly dissimilar materials such as the compliant tendon to the much stiffer bone, over a very small region. The first aim of this review is to highlight mathematical and computational models that have been developed to investigate the many attachment strategies present at entheses at different length scales. Entheses are also relevant in the medical context due to the high prevalence of orthopedic injuries requiring the reattachment of tendons or ligaments to bone, which are associated with a rather poor long-term clinical outcome. The second aim of the review is to report on the computational works analyzing the whole tendon to bone complex as well as targeting orthopedic relevant issues. Modeling approaches have provided important insights on anchoring mechanisms and surgical repair strategies, that would not have been revealed with experiments alone. We intend to demonstrate the necessity of including, in future models, an enriched description of enthesis biomechanical behavior in order to unravel additional mechanical cues underlying the development, the functioning and the maintaining of such a complex biological interface as well as to enhance the development of novel biomimetic adhesive, attachment procedures or tissue engineered implants.

## Highlights

• Entheses solve the challenging task of connecting dissimilar tissues over a tiny region.
• Several key features facilitate load transmission from soft tissues to bone.
• The role of modeling and simulations for investigating entheses is highlighted.
• Models allows to link enthesis microstructure and composition to mechanical properties.
• Computer simulations at the organ scale answer orthopedic relevant questions on enthesis failure.

## 1 Introduction

**Fig. 1:**
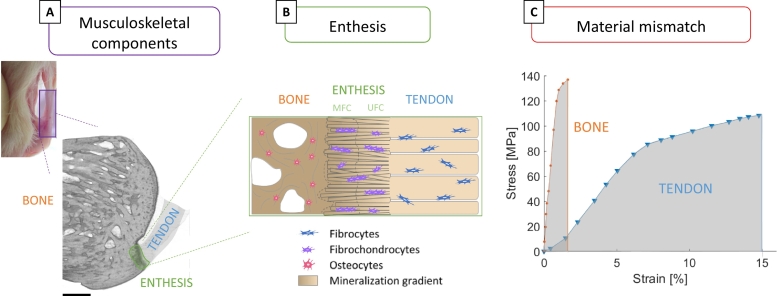
(A) Micro-computed tomography (micro-CT) reconstruction of a calcaneus bone-tendon complex (rat) highlighting the location of the enthesis at the posterior side of the bone. Scale bar: 500 μm. (B) Schematic representation of the enthesis at the tendon to bone insertion comprising unmineralized fibrocartilage (UFC) and mineralized fibrocartilage (MFC). (C) Typical tissue level stress-strain behavior of bone and tendon, underlining mismatches in stiffness (bone is stiffer than tendon) and toughness (tendon is tougher than bone) that should be “resolved” at the interface. Figures modified from (Wagermaier et al., 2015; Nourissat et al., 2010), with permission.

The musculoskeletal system comprises not only different tissues such as muscle, tendon, ligament, cartilage and bone, serving diverse biological and biomechanical functions, but also many internal interfaces joining them. Although interfaces occupy only a small fraction of the overall tissue volume, they are crucial for the structural integrity and functioning of the musculoskeletal apparatus (Dunlop et al., 2011). Interfaces are specialized regions facilitating load transmission between dissimilar tissues, which is a challenging task when the tissues being joined have a large mismatch in constitutive material behavior. From a mechanical viewpoint, a sharp transition in material properties can give rise to local high stresses at bimaterial junctions (Desmorat and Leckie, 1998). As a consequence, upon repeated loading, contact failure at a level of stress lower than the strength of the two materials may happen (Liu et al., 2011). In contrast with this well-accepted mechanical phenomenon, healthy musculoskeletal interfaces have the remarkable ability to sustain large forces (even higher than body weight) for several millions of loading cycles. This is the case of the interface anchoring tendon (or ligament) to bone, called enthesis (Fig. 1A). The tissues being joined at entheses have distinct composition, structure and biomechanical function (Fig. 1B) (Spalazzi et al., 2013). Tendons are effective biological springs designed to sustain large tensile stresses: they have to be stiff enough to prevent excessive stretching when loaded by the muscles, but also somewhat compliant to bend around bones and to allow changes in joint position (Biewener, 2008). At the tissue level, the stress-strain curve of tendons is non-linear, with a characteristic J-shape where the stiffness increases with strain up to a Young's modulus of about 0.5–1.5 GPa (Fig. 1C). Although tendons show a heterogeneous mechanical response, strongly depending on the anatomical location and health status, they can be considered both fairly strong (tensile strength from 50 to 150 MPa) and extensible (failure strains up to 12–15%) (Martin et al., 1998). The combination of these two properties results in a material with high toughness, which is approximately defined as the ability to absorb energy before failure. On the other side, bones have to face a more complex loading scenario featuring tensile, compressive, and shear stresses. They have to be very stiff to prevent excessive deformation under load but also as tough as possible to avoid fractures (Currey, 2002). At the tissue level, bones have a different stress-strain behavior than tendons (Fig. 1C): they are much stiffer (Young's modulus of 15–25 GPa), roughly equally strong (tensile strength of 120–180 MPa) but much less deformable (failure strains of about 2–3%), resulting in smaller toughness. Connecting two tissues with such dissimilar mechanical characteristics is not an easy task, as mismatches in stiffness, deformation mode and toughness can trigger interface failure (Fan et al., 2015; Cordisco et al., 2012; Thomopoulos et al., 2013). Indeed, despite an extraordinary endurance, entheses are vulnerable to injuries (Shaw and Benjamin, 2007), which may need surgical interventions. Rotator cuff tear is a frequent orthopedic problem occurring typically at the tendon insertion and often requiring the surgical reattachment of the soft tissue to the bone (Teunis et al., 2014; Gianotti et al., 2009; Patel et al., 2018; Rossi et al., 2019). The rupture of the anterior cruciate ligament (ACL) is another common injury: even if ACL breaks more frequently at the mid-substance rather than at the enthesis, the ACL is often entirely replaced using grafts (ACL reconstruction), which need to be anchored to the bone and which require proper soft tissue to bone healing (Lyman et al., 2009; Bedi et al., 2010). There are many indications that the long term clinical outcome of reattachment and de novo anchoring procedures can be rather poor (Bedi et al., 2010), one reason being the lack of proper enthesis regeneration and healing.

In contrast to other biological systems where dissimilar materials are joined using compositional gradients over a length scale of several millimeters (e.g., squid beak and byssus threads), which is much larger than the micrometer-size of the elementary building blocks (Boys et al., 2019; Miserez et al., 2008; Harrington and Waite, 2009), musculoskeletal tissues such as tendon and bone are attached over a very tiny transition region, spanning only a few hundreds of micrometers (Spalazzi et al., 2013). This may require the combination of multiple mechanisms to provide entheses with adequate mechanical competence and failure resistance. Indeed, entheses are complex biological interfaces which appear either as fibrous or fibrocartilaginous, depending on the anatomical location and corresponding mechanical requirements (Benjamin et al., 2002). In fibrous insertions, the soft tissue attaches to bone in a direct manner and in the presence of substantial fibrous connective tissue, as for example at the insertion between medial collateral ligament and tibia, or deltoid tendon and humeral head (Apostolakos et al., 2014). Fibrocartilaginous entheses are more structured and have higher clinical relevance, as they are found at sites suffering from enthesis injuries and pathologies (Lu and Thomopoulos, 2013; Benjamin et al., 2006). In short, fibrocartilaginous entheses traditionally comprise four adjacent tissues (Fig. 1B): tendon (or ligament), unmineralized fibrocartilage (UFC), mineralized fibrocartilage (MFC) and bone. Tendons and ligaments have a highly ordered and aligned hierarchical structure, based on collagen type I molecules assembling in a staggered manner to form fibrils, which are then arranged in higher level structural patterns such as fibers and fascicles (Fratzl and Weinkamer, 2007). The cells responsible for the synthesis of the fibrous collagen-based matrix in this region are fibroblasts. The next region is UFC, which lacks the long range order of tendons and ligaments, but still presents parallel arrays of collagen fibrils embedded into a more random fibrous network (Rufai et al., 1996; Benjamin and Ralphs, 1998). Fibrocartilage is mainly composed of collagen type I, II and III, together with aggrecan, a relatively large proteoglycan molecule. Before reaching bone, fibrocartilage gets reinforced by nanometer-sized mineral particles (Sinclair et al., 2011), and features an organic matrix mainly composed of collagen type II and X together with other cartilage-associated proteins (Lu and Thomopoulos, 2013; Waggett et al., 1998). In histological sections, the interface between unmineralized and mineralized cartilage is clearly demarcated by a “tidemark”, which coincides with the mineralization front. The main cells in charge of synthetizing the extracellular matrix of fibrocartilage are fibrochondrocytes. From a mechanobiological point of view, the presence of fibrocartilage at entheses is believed to be an adaptation to compressive loading (Benjamin and Ralphs, 1998). The last zone is bone, with the basic building block being the mineralized collagen fibril, forming parallel arrays which are then arranged in different structural patterns at higher level of the hierarchical ladder (Weinkamer and Fratzl, 2016). In bone, the cells encased within the mineralized extracellular matrix are the osteocytes, which are kept alive and communicate with each other thanks to an intricate canalicular network (Weinkamer et al., 2019; Buenzli and Sims, 2015). The transitions in composition, mineralization and structural arrangement among the four regions are believed to occur in a gradual manner, which is a basic requirement to avoid dangerous stress localization. Nevertheless, such gradients are restricted to the micrometer length scale and the identification of additional strengthening and toughening strategies present at enthesis is a crucial step (Rossetti et al., 2017), not only to improve the understanding of the healthy tendon to bone insertion (Moffat et al., 2008), but also to develop new approaches for treating enthesis injuries and ruptures (Lu and Thomopoulos, 2013). There are several excellent studies focusing on different aspects of entheses: a good starting point are the works of Benjamin and colleagues, drawing the attention to the importance of entheses as complex “organs” at musculoskeletal interfaces and their related clinical disorders (Benjamin et al., 2002; Benjamin et al., 2006; Benjamin and Ralphs, 1998). Thomopoulos and co-workers have performed extensive research on enthesis biomechanics, mechanobiology, development and healing, including the effects of unloading, well summarized in several papers (Liu et al., 2011; Thomopoulos et al., 2013; Lu and Thomopoulos, 2013; Schwartz and Thomopoulos, 2013; Derwin et al., 2018; Deymier et al., 2019). Mechanoregulated molecular aspects of enthesis development have been also investigated and reviewed by Zelzer and colleagues (Felsenthal and Zelzer, 2017; Zelzer et al., 2014). Various imaging modalities are available to characterize such a multi-tissue region at different length scales, both in the clinical context as well as in basic research, as discussed in (Bannerman et al., 2014). A currently active research route is about repair and tissue engineering options for orthopedic interfaces, as presented in numerous reviews (Patel et al., 2018; Bonnevie and Mauck, 2018; Boys et al., 2017; Locke et al., 2017; Lee et al., 2016; Calejo et al., 2019). Together with experimental approaches, modeling and simulations have been used as complementary tools to elucidate the structure-function relationship at entheses. The main goal of the present review is to summarize mathematical and computational approaches that have been used, in close synergy with experiments, to characterize the biomechanical behavior of entheses at different hierarchical levels. We classify the modeling approaches in two categories: models which have been used to highlight, at specific length scales, the contribution of microstructural and compositional adaptations to improve anchoring properties (Section 2), and models of the whole soft tissue to bone complex which have been developed to evaluate overall load transfer mechanisms from soft tissue to bone as well as to answer orthopedic questions (Section 3). The review shall illustrate how the knowledge of the local mechanical environment can improve our understanding of this complex multi-tissue region as well as suggest new aspects deserving more consideration in future studies.

## 2 How to link microstructure and composition to mechanical properties?

This section presents modeling strategies which have been employed to investigate the mechanical role of specific compositional and microstructural features experimentally observed at entheses. Computational models, although idealized, allow the separation of the relative contributions of compositional and microstructural adaptations on the local mechanical behavior. Bones and tendons are both fibrous composite materials (Fratzl and Weinkamer, 2007): going from tendon to bone, the focus is first on the spatial variations in fiber architecture (Section 2.1). Then, the impact of mineral reinforcements - which appear starting from the transition region between UFC and MFC - is analyzed, highlighting the effect of reinforcements at different length scales (Section 2.2). Surface roughness and interdigitations are present at the interface between UFC and MFC and also between MFC and bone. In Section 2.3, their biomechanical role is reviewed. Major insights and perspectives regarding those three main aspects are summarized in Table 1. In the present review, if not stated otherwise, we use collagen fibril to indicate a collection of self-assembled tropocollagen molecules, having diameter at the nanometer length scale (typical values reaching a few hundreds of nanometers) (Biewener, 2008). With collagen fiber, we indicate an assembly of several fibrils with an indicative final diameter up to several tens of micrometers.

**Table 1:** Linking microstructure and composition to mechanical properties.

Highlights
• The fibrous nature of tendon is preserved across the interface, with tendon **fibers splaying** and **unravelling** to anchor to bone. This generates a **spatial variation in fiber architecture and orientation**, with the degree of alignment decreasing when approaching bone. • Collagen fibers are **progressively reinforced by mineral crystals** when crossing the interface between unmineralized and mineralized fibrocartilage. This reinforcement provides substantial stiffening to fibers only for mineral accumulation exceeding a **percolation threshold**. • The complex interplay between fiber orientation and degree of mineralization results in a **non-monotonic variation of tissue stiffness** along the insertion, with the appearance of a **compliant region** having stiffness lower than tendon and bone. • Interface **roughness** and **interlocking** between different sub-regions of the enthesis help to **increase fracture toughness**.
Limitations and outlooks
• The fibrous nature of the insertion suggests that discrete network models accounting for the individual collagen fibers could provide new insights into enthesis biomechanics, for example by elucidating the link between fiber architecture and local damage mechanisms at the sub-tissue scale. • In addition to tissue stiffness, the spatial variation of other material properties such as viscoelasticity and strength should be characterized and interpreted based on fiber architecture and degree of mineralization. • The role of internal interfaces and their complex interlocking patterns should be further investigated. A computational model comprising the interface between unmineralized and mineralized fibrocartilage and the one between mineralized fibrocartilage and bone could unravel new avenues to improve enthesis fracture toughness.

### 2.1 Fiber architecture

Despite being based on the same type of collagen, an important difference between tendon and bone – in addition to the presence of mineral - is the arrangement of the collagen fibers. In tendon, fibers are mainly parallel and fairly well aligned along a common direction, usually corresponding to the main axis of the tendon (Fratzl, 2008), with the degree of alignment even increasing when the tendon is loaded (Fratzl and Weinkamer, 2007). In bone, the orientation of the mineralized collagen fibers is more sophisticated, probably reflecting more complex mechanical requirements. Considering lamellar bone, for example, fibers can form either rotated plywood structures as in cortical bone (Wagermaier et al., 2006) or parallel arrays following the local predominant orientation of trabeculae as in cancellous bone (Reznikov et al., 2015). Common approaches to characterize quantitatively the orientation of collagen include: polarized light microscopy (Bromage et al., 2003; Thomopoulos et al., 2003), polarized Raman and Fourier-transform infrared spectroscopy (FTIR) (Galvis et al., 2013; Kazanci et al., 2006; Bi et al., 2005), as well as x-ray-based techniques such as small angle x-ray diffraction (Paris et al., 2000; Boote et al., 2004). However, these methods are mainly restricted to two-dimension and current research is focusing on new strategies to derive orientation in three-dimension (Georgiadis et al., 2016).

**Fig. 2:**
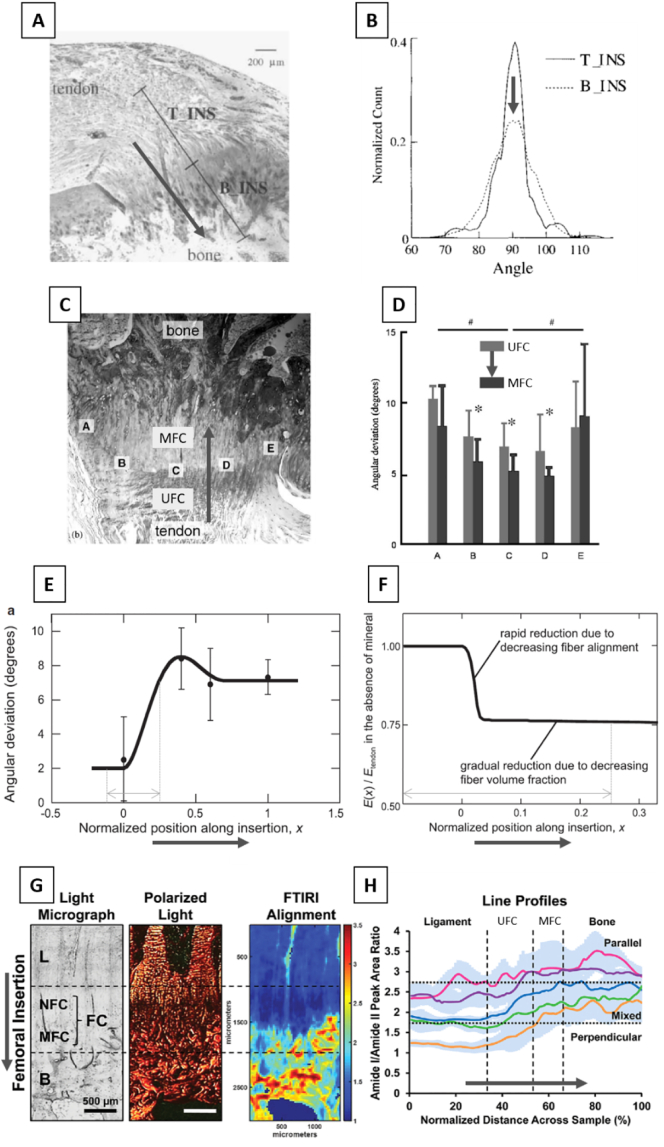
(A) Histological cross-section of the supraspinatus tendon to bone insertion (rat sample) showing the two regions used to measure orientation of collagen fibers with polarized light (T-INS: tendon end of the insertion, B-INS: bone end of the insertion). (B) Bell-like frequency distribution of collagen fibers orientation for the tendon insertion (T-INS) and the bony insertion (B-INS). Note that the 90° orientation lies along the main axis of the tendon. The distribution at the bone insertion shows a slightly higher heterogeneity. (C) Bright field microscopy of the supraspinatus insertion showing the regions (A–E) used for collagen orientation assessment on both sides of the tidemark (unmineralized fibrocartilage, UFC and mineralized fibrocartilage, MFC). (D) Resulting angular deviation measured with polarized light showing that fibers in the center (B–D) are more organized at the MFC compared to the UFC. (E) Spatial variation of angular deviation (which defines how well the fibers are aligned along a common direction) across the tendon (position 0) to bone (position 1) interface. Smaller values indicate higher alignment. (F) Spatial variation in elastic modulus computed with a continuum mechanics approach assuming the angular dispersion visualized in (C) and an ideal enthesis with no mineral. Decreasing fiber alignment causes a sudden and substantial drop in modulus. (G) Organization of collagen fibers across the anterior cruciate ligament (ACL) to bone insertion. Light micrograph (left) with the different tissues encounter at the enthesis (L: ligament, NFC: non-MFC, B: bone). Polarized light images (middle) of sections stained with Picrosirius red to highlight collagen arrangement and (left) corresponding FTIR spectroscopic maps (fiber orientation is color-coded as follows: blue perpendicular to interface; yellow: mixed orientation; red: parallel to interface). (H) Line profile analyses of spectroscopic maps for five samples (full line is the mean and shaded area shows standard deviation for each sample) revealing a clear transition in fiber organization. Bold grey arrows represent the tendon to bone direction. Figures modified from (Thomopoulos et al., 2003; Qu et al., 2017; Thomopoulos et al., 2006; Genin et al., 2009), with permission.

The spatial arrangement of collagen fibers at the interface between bone and soft tissues largely depends on the anatomical location and on the type of interface considered. At the interface between bone and articular cartilage, an abrupt change in fiber orientation has been reported (using x-ray scattering), with fibers arranged parallel to the interface from the bone side and perpendicular to the interface from the cartilage side (Zizak et al., 2003). Such arrangement is thought to favor a tight connection between bone and articular cartilage, preventing cartilage failure by reducing its later extension (Zizak et al., 2003). Conversely, at the connection between the supraspinatus tendon and the humerus (Fig. 2A), fibers tend to have a similar predominant direction (along the major axis of the tendon) which is maintained after crossing the interface, as measured with polarized light (Thomopoulos et al., 2003). However, at the bony insertion, the frequency distribution of fiber orientation showed somewhat higher heterogeneity when compared to the tendon insertion (Fig. 2B), suggesting a less ordered arrangement. This observation was refined in another study focusing on the same anatomical location (i.e. shoulder joint). The angular deviation of fiber orientation, which is an indication of how well the fibers are aligned along a common direction, was measured with polarized light at adjacent sides along the tidemark between MFC and UFC (Fig. 2C, positions A to E), with the mineralized side of the tidemark showing significantly smaller angular deviation (Fig. 2D). The information of both studies on the rotator cuff were used to extrapolate the spatial evolution of the angular deviation from tendon to bone (Fig. 2E), by combining measurements done within tendon and bone (Fig. 2B) with measurements performed along the tidemark (Fig. 2C) (Zizak et al., 2003). Going from tendon to bone, the angular deviation increased of almost a factor of four, and reached a small peak before hitting the tidemark between UFC and MFC, hinting for a disordered region before entering into the mineralized tissue. Considering a different anatomical location (the knee joint) and a ligament-bone interface, high resolution two-dimensional (2D) spatial mapping of fiber orientation have been obtained at the femoral and tibial insertions of ACL combining polarized light and FTIR (Spalazzi et al., 2013; Qu et al., 2017). Results revealed a quite heterogeneous arrangement (as estimated by the area ratio of Amide *I* and *II* peak in the spectra) with collagen fibers in the ligament oriented predominantly perpendicular to the interface but changing the orientation when crossing fibrocartilage region and reaching bone with a more oblique organization (Fig. 2G and H). Overall, those studies suggest that collagen fibers are locally organized into parallel bundles also within fibrocartilage, corroborating previous qualitative observations based on electron microscopy at the Achilles tendon enthesis (Rufai et al., 1996), but most likely being less ordered than in tendon or bone.

Collagen fibers (and fibrils) are long and thin structural elements and, in the absence of mineral reinforcements, they are usually strong in tension and weak in compression (Fratzl and Weinkamer, 2007). Therefore, small changes in fiber orientation can have a large impact on the mechanical behavior. Indeed, the modulation of fiber orientation is a well-known approach used both in biological (Wagermaier et al., 2006; Daxer and Fratzl, 1997) and in synthetic fibrous composites (Zorzetto and Ruffoni, 2019; Hull and Clyne, n.d.), to finely tune the local mechanical behavior without modifying chemical composition. There are numerous experimental assessments of fiber organization at the soft tissue to bone interface (Spalazzi et al., 2013; Thomopoulos et al., 2003; Zizak et al., 2003; Qu et al., 2017; Genin et al., 2009); however, one challenge of the experimental works is that when crossing the interface between UFC and MFC, collagen gets reinforced with mineral crystals (as presented in the next section). It is therefore difficult to isolate the contribution of fibers orientation on the overall mechanical competence. This challenge can be overcome thanks to computational approaches. Essentially, there are two main modeling strategies to link fiber arrangement with mechanical behavior: classical continuum structural mechanics approaches and discrete fiber network models. In the continuum mechanics frameworks (Thomopoulos et al., 2006; Zickler et al., 2012; Gasser et al., 2006), fibers are not explicitly modeled; instead, at each location, an elasticity tensor linking tissue stress with strain is derived based on averaged and simplified descriptors of fiber mechanical behavior, packing and orientation (Gasser et al., 2006). Usually, these models are computationally inexpensive and can capture fairly well the overall stress-strain behavior at the tissue level. The obtained constitutive relations can then be implemented into continuum level finite element (FE) models (Ruffoni and van Lenthe, 2017), therefore accounting for more complex geometries and heterogeneous tissues (Thomopoulos et al., 2006; Gasser et al., 2006). However, continuum approaches cannot resolve mechanical events at the level of individual fibers (or at the fiber-matrix interface). Such information could be essential to understand the interaction between the fiber network and other elements such as cells and interfibrillar matrix or to account for failure at discrete attachment points where collagen fibers (or fiber bundles) anchor to bone (Rossetti et al., 2017). Individual collagen fibers are explicitly considered in discrete fiber network approaches (Wang and Ural, 2018; Dong et al., 2017; Lee et al., 2014; Ban et al., 2019; Wang et al., 2014; Zhang et al., 2012; Richardson et al., 2018), which are computationally more demanding than continuum models and, therefore, smaller volumes of interest and length scales are usually investigated. Discrete fiber models have been adopted to characterize force transmission in collagen-based networks, and allow capturing complex coupling mechanisms among different deformation modes (Ban et al., 2019), which are not emerging when using continuum models. They are also useful in the presence of substantial reconfiguration of the network under load (Wang et al., 2014). In the bone context, discrete fiber models have been used to investigate the link between the spatial organization and the fracture behavior of mineralized collagen fibrils (Wang and Ural, 2018; De Falco et al., 2017). In contrast to the many computational works dealing with fibril arrangement in collagen-based tissues, models devoted to the investigation of fiber properties at the soft tissue to bone insertion are still rather sparse (Liu et al., 2011; Thomopoulos et al., 2006; Genin et al., 2009). The micromechanical consequences of the spatial variation in fiber orientation across the bone-tendon interface (at the shoulder joint) have been estimated using a continuum mechanics unit cell model, with fibers idealized as linear elastic transversally isotropic elements, together with additional assumptions on mineral accumulation within fibers and fiber three-dimensional (3D) orientation (Genin et al., 2009). The local tissue elastic modulus was computed based on the experimentally assessed angular deviation of fiber orientation (Fig. 2E) and mineral volume fraction, at different spatial positions across the interface. Assuming a hypothetical enthesis without mineral, the elastic modulus showed a fairly large drop (about 25%) as soon as the angular deviation increased above 2.5 degrees (Fig. 2F). This behavior can be mechanically justified by a switching in the main deformation mode of the fibers from stretching to bending as the angular deviation increases.

**Fig. 3:**
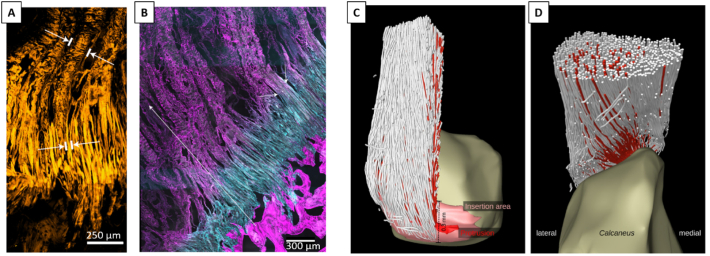
(A) Confocal microscopy image of tendon fibers unravelling into thinner interface fibers before attaching to bone. The white marks indicate fiber width. (B) Cryo-cut immunostained section imaged with confocal microscopy showing collagen type I (magenta) and II (cyan) fibers. Fiber composition changes within a ~500 μm thick region preceding bone. The transition in fiber composition coincides with the transition in fiber architecture (splaying and unravelling) as highlighted by the short white arrows. (C) and (D) show two different perspectives (posterior and superior) of the fibrous collagen network visualized in 3D using propagation-based phase-contrast micro-CT, which allows resolving and tracking the path of individual collagen fibers. The red color indicates fibers inserting into a specific region (protrusion) of the calcaneus bone. Figures modified from (Rossetti et al., 2017; Sartori and Stark, 2020), with permission.

To the best of our knowledge, there are no studies using discrete network models to characterize anchoring mechanisms at musculoskeletal interfaces. Nevertheless, recent experimental works have highlighted additional features of the fibrous collagen network at enthesis (Rossetti et al., 2017; Sartori and Stark, 2020; Sartori et al., 2018), which could be investigated with such approach. Rossetti and coworkers (Rossetti et al., 2017), using high resolution micro-computed tomography (micro-CT), confocal imaging and electron microscopy, revealed that tendon fibers “split” into an almost order of magnitude thinner interface fibers before inserting into calcaneus bone (Fig. 3A). The decrease in fiber diameter is accompanied by a splay out of interface fibers, with the anchoring to bone eventually taking place at discrete locations along a wavy interface. The transition in fibers geometry coincide with a switch in network composition from collagen type I to type II, as evidenced by confocal imaging of immunostained sections (Fig. 3B). From a mechanical viewpoint, the unravelling and splaying out of tendon fibers are advantageous mechanisms as they contribute to decrease stress concentration (by increasing attachment area) as well as stress edge singularities (by reducing the angle of force transmission at the interface) (Liu et al., 2011; Balijepalli et al., 2016; Deymier-Black et al., 2015). Furthermore, in situ mechanical testings, combined with multiscale confocal microscopy and advanced image processing to map the displacement field, revealed that at the interface, the group of fibers involved in the transmission of load from tendon to bone changes with the angle of force application. This phenomenon leads to an angle-dependent force redistribution, with dedicated fibers recruited at different stages and supporting different types of loads (e.g. shear, buckling or in-plane rotations), with the overall effect of increasing interface resilience (Rossetti et al., 2017). Others have highlighted the 3D arrangement and the topology of the collagen network at the insertion between Achilles tendon and calcaneus bone using propagation-based phase-contrast micro-CT in combination with a fiber tracking algorithm, even resolving individual collagen fibers (Sartori and Stark, 2020). The study confirmed a smaller fiber cross-sectional area at the enthesis and revealed detailed aspects of fiber curving, branching and twisting before entering into bone (Fig. 3C and D). This novel microstructural information should be cast into computational models accounting for individual collagen fibers to elucidate their impact on the biomechanical behavior of the enthesis.

### 2.2 Mineral reinforcements

**Fig. 4:**
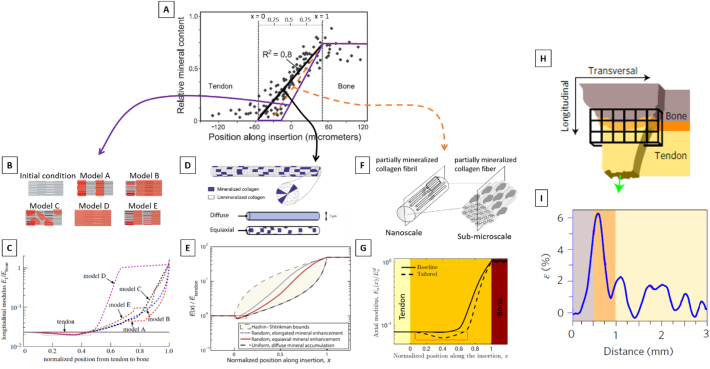
(A) Relative mineral content (estimated using Raman spectroscopy) across the tendon to bone insertion. The gradual increase in the mineral content occurs over a 120 μm thick region and is well fitted by a linear function. (B) Micromechanical model of the mineralized collagen fibril at the sub-micrometer length scale with hypothetical scenarios for the mineralization process. (C) Corresponding spatial modulation of the longitudinal modulus along the tendon-bone interface, obtained considering a gradient starting at *x* ~ 0.35 (purple line in A, *x* denotes the normalized position along the tendon-bone interface). Depending on the details of mineral nucleation and accumulation, different elasticity gradients could be derived. (D) Micromechanical model of a mineralized collagen fiber at the micrometer length scale, used to compute the elastic properties variation from tendon to bone assuming three different scenarios for the mineral distribution within the fiber. (E) Corresponding normalized modulus variation from tendon to bone, obtained considering a gradient starting at *x* ~ 0 (black line in A): a small compliant zone with stiffness lower than tendon and bone is visible. (F) Representation of two specific steps of a multiscale approach, illustrating a partially mineralized collagen fibril and fiber. (G) Corresponding normalized axial modulus variation from tendon to bone. The full line is obtained considering a gradient starting in x ~ 0, whereas the dashed line a gradient starting in x ~ 0.2 (i.e., orange line in A). In agreement with previous models, the spatial delay in mineralization front induces a compliant region (highlighted by a red frame). (H) Micromechanical testing of enthesis samples (green arrow shows the applied deformation along the longitudinal direction). (I) Strain values at the enthesis: the high initial peak of the strain corresponds to a locally softer material. Figures modified from (Rossetti et al., 2017; Genin et al., 2009; Liu et al., 2013; Aghaei et al., 2020), with permission.

In mineralized tissues such as bone and fibrocartilage, the collagen fibrils are reinforced by nanometer-sized mineral crystals (Fratzl and Weinkamer, 2007). The relative arrangement between collagen and mineral is of paramount importance for the mechanical behavior of the mineralized collagen fibril. In mature bone, stiff mineral crystals are arranged in a staggered fashion within the collagenous matrix, with force transmitted to the mineral particles through shearing of the matrix, when the fibril is loaded in tension (Jäger and Fratzl, 2000). This mechanism is a universal construction principle common to other biological materials (Ji and Gao, 2004), which provides the composite with high stiffness (from the mineral) and high toughness (from the matrix) (Fratzl and Weinkamer, 2007; Gao et al., 2003). Noteworthy, the same staggered arrangement between stiff and compliant building blocks is repeated at higher structural length scales in both bone and tendon, and has a profound influence on the deformation mechanisms (Gupta et al., 2006). The local distribution of mineral within a calcified tissue can be quantified experimentally with various techniques, the most common being: quantitative backscattering electron imaging (qBEI) (Roschger et al., 2008; Lukas et al., 2011a), Raman and FTIR (Kazanci et al., 2006; Paschalis et al., 2011), synchrotron radiation microcomputed tomography (Nuzzo et al., 2002; Borah et al., 2006) and, to some extent, desktop micro-CT (being aware of beam hardening artifacts which can alter the results) (Lukas et al., 2011b; Mashiatulla et al., 2017; Bounoure et al., 2014). As a consequence of the biological processes of bone remodeling and mineralization, bone tissue is not uniformly mineralized but shows a characteristic distribution of mineral content, which can be analyzed with the help of mathematical models to extract information on the kinetics of mineral incorporation into the organic matrix in both trabecular (Ruffoni et al., 2007; Ruffoni et al., 2008) as well as cortical (Lerebours et al., 2020) bone. Interestingly, although both tissues are made up of lamellar bone, the mineralization (and therefore the stiffening) process seems to proceed at distinct velocities, perhaps due to more complex microstructural differences than commonly believed (Lerebours et al., 2020). Regarding entheses, mineral content and heterogeneity of MFC have been measured across multiple species and locations (Spalazzi et al., 2013; Sinclair et al., 2011; Moffat et al., 2008; Qu et al., 2017; Shea et al., 2002; Ferguson et al., 2003); however, details of the mineralization process of this tissue are less clear (Schwartz et al., 2012; Schwartz et al., 2015; Liu et al., 2013). At the interface between unmineralized tissues and bone, the mineral content has to increase from 0% to about 50% volume fraction and, consequently, biomechanically crucial questions that should be answered are: *i*) what is the width of the transition region between unmineralized and mineralized tissue? *ii*) how does the mineral content vary across this region? and *iii*) what is the corresponding behavior of the local mechanical properties (e.g. elastic modulus) in this region? The width of the transition zone between UFC and MFC seems to be very tiny and, depending on species, anatomical location and assessment technique, ranges from about 10 to 120 μm (Spalazzi et al., 2013; Deymier-Black et al., 2015; Schwartz et al., 2012; Wopenka et al., 2008; Deymier et al., 2017). A slightly larger width (~190 μm) has been observed at meniscal attachments (Boys et al., 2019), whereas at the bone-articular cartilage interface the mineral content shows a strong increase within a zone of only 20–40 μm (Gupta et al., 2005); others have even described a surprising smaller transition region, as small as 5 μm (Campbell et al., 2012). Considering the variation of mineral content across the interface, a pioneering work of Wopenka and colleagues described a fairly linear gradient at the mature rat supraspinatus to bone interface (Fig. 4A) (Wopenka et al., 2008), while subsequent investigations point towards steeper exponential (or even step-wise) variations, as measured at the ACL tibial and femoral insertion (Spalazzi et al., 2013; Qu et al., 2017). Analyzing developing entheses, the gradient between calcified and uncalcified enthesis seems to be present even before the appearance of fibrocartilage and, therefore, could be an intrinsic feature of the mineralization front associated with endochondral ossification (Schwartz et al., 2012). Nevertheless, different spatial increase in mineral content from soft tissue to bone may also underline dissimilarities in the biomechanical requirements of the interfaces. Finally, the connection of mineral gradients (and tissue composition) to local mechanical behavior is probably the most challenging question to answer. Experimentally, spatial mapping of local tissue elasticity at musculoskeletal interfaces has been obtained with nanoindentation (Ferguson et al., 2003; Gupta et al., 2005; Hauch et al., 2009), atomic force microscopy (Campbell et al., 2012), confocal elastography (Boys et al., 2019), scanning acoustic microscopy (Sano et al., 2006a) and micromechanical testing (Rossetti et al., 2017; Moffat et al., 2008; Deymier et al., 2017; Sevick et al., 2018). In some cases, the mechanical information has been directly correlated with the mineral content (Ferguson et al., 2003; Gupta et al., 2005). Despite the compositional and microstructural complexity of the enthesis, a gradient in the mineral content engenders a fairly similar graded transition in local elastic properties (Ferguson et al., 2003; Campbell et al., 2012); at the same time, viscous damping has an opposite trend and seems to vary across a broader region (Campbell et al., 2012; Abraham and Haut Donahue, 2013). Less evident is that the correlation between local stiffness and mineral content can be different in mineralized cartilage compared to bone. Indeed, mineralized (articular) cartilage requires a higher mineral content than bone to reach the same Young's modulus, most likely due to differences in the organic matrix (Gupta et al., 2005). Whether this is also the case for MFC is not known; however, a better understanding of the material behavior of MFC would be relevant, for example to improve the modeling of the deformation and fracture of such tissue.

A number of modeling and computational studies have characterized the role of mineral reinforcements at the soft tissue-bone interface. As nanoindentation is one of the most straightforward experimental approach to characterize the mechanical behavior of multi-tissue interfaces, it is worth mentioning a combined experimental-computational work evaluating the ability of such technique to measure mechanical properties across two highly dissimilar materials (Armitage and Oyen, 2017). FE models where used to investigate the relationship between the measured and the real spatial distribution of the elastic modulus across a graded bimaterial interface. The authors provided general guidelines to investigate mechanical properties variation in tissues with significant modulus heterogeneity, by demonstrating that the indenter size should be less than 10% of the expected length scale of the modulus variations. Translated to the context of interfaces between soft tissue and bone, assuming an interface width of 20 μm, current nanoindentation techniques should use probes smaller than 2 μm, which could make the quantification of the properties of tissues with stiffness of the order 10–100 MPa quite challenging (Armitage and Oyen, 2017). This could be problematic not so much for the bone-tendon interface but more for bone-cartilage or bone-meniscal junctions.

Focusing on modeling studies, a number of micromechanical models have been developed for answering relevant research questions on *i*) the impact of mineral reinforcements on the biomechanical behavior of collagen at different length scales (from nano up to tissue level), *ii*) the role of gradients in mineral content on the elastic properties of partially mineralized collagen fibrils and fibers, and *iii*) the interplay between mineral reinforcements and fiber architecture for the overall tissue level properties across the entheses (Genin et al., 2009; Liu et al., 2013; Aghaei et al., 2020; Saadat et al., 2015). One first fiber-level model was developed to predict the impact of mineral gradients on the local stiffening effect in partially mineralized collagen fiber (Genin et al., 2009). The model assumed different idealized relative arrangements of clusters of mineralized and unmineralized collagen at the micrometer length scale, including a “diffuse” model describing uniform mineralization, an “equiaxial” model representing non-uniform mineral accumulation and an “elongated” model where mineral clusters were non-uniformly distributed and also geometrically elongated along the fiber direction (Fig. 4D). The model was solved using a continuum mechanics framework in combination with FE analysis, and allowed computing the full tissue level stiffness tensor. At the percolation threshold, corresponding to the formation of a large uninterrupted cluster of mineral spanning the entire length of the collagen fiber, the stiffening effect of the mineral reinforcements increased substantially. The different mineral arrangements had an impact on the degree of mineralization needed to reach the percolation threshold. To better capture the mechanisms of mineral accumulation at sub-micrometer length scales, Liu and co-workers proposed a framework for partially mineralized collagen fibrils accounting for differences in the initiation and sequence of mineralization (Liu et al., 2013). Specifically, five distinct scenarios were considered, starting from a completely unmineralized collagen fibril (Fig. 4B). Model A: mineralization initiated at the gap region followed by extrafibrillar mineralization nucleating over the gap regions and spreading along the fibril; model B: like model A but extrafibrillar mineralization started from a single nucleation site on each fibril; model C: same as models A and B concerning initiation within the gap region, followed by random accumulation of mineral at the exterior of fibrils, with no subsequent growth of these accumulations; model D: random extrafibrillar accumulations of mineral preceding the mineralization of gap regions; model E: identical to model A except that it included mineralization of the overlap regions following the filling of the gap regions and prior to extrafibrillar mineralization. The (partially) mineralized fibrils were then included into higher hierarchical level fibers using additional assumptions and finite-element (FE) simulations. In addition to confirming the percolation threshold as a key factor for fiber stiffening, those models revealed how the specific sequence of mineralization could impact the degree of tissue stiffening: mineralization models which delayed the percolation of the mineral phase lead to a “slower” spatial gradient in tissue modulus. The collagen-mineral biocomposite has also been investigated in more general terms, considering micromechanical approaches developed for man-made composite materials containing multiple inclusions (Aghaei et al., 2020; Saadat et al., 2015). This route was followed to estimate the elastic properties of an idealized soft collagen matrix reinforced by a high volume fraction of stiff mineral in the form of ellipsoidal inclusions (Saadat et al., 2015). The model was used to derive longitudinal and transverse elastic moduli of partially mineralized collagen fibers as a function of mineral content and showed good agreement with (computationally more expensive) approaches based on FE analysis (Liu et al., 2013). In a similar context, a multiscale model based on homogenization procedures has been developed to include the contribution of several nano-, sub-micro- and microstructural features observed at the interface, on the effective anisotropic stiffness tensor of the tissue (Fig. 4F) (Aghaei et al., 2020). The study, although based on different assumptions and following an alternative methodology, confirms the presence of a percolation threshold above which mineral accumulation within collagen can provide substantial stiffening.

Using some of the presented modeling strategies, the gradual transitions in mineral content can be combined with the reported angular deviation of the collagen fibers to derive the corresponding spatial variation of the overall tissue level elastic behavior across the interface (Genin et al., 2009; Liu et al., 2013; Aghaei et al., 2020). Interestingly, all these studies have found a non-monotonic variation in tissue stiffness across the insertion, highlighting a compliant region with stiffness lower than both tendon and bone (Fig. 4C, E and G). Such counterintuitive behavior can be explained by the interplay between fiber alignment (which is reduced approaching the interface) and mineralization (which gradually increases and shows a significant contribution above the percolation threshold). Noteworthy, a compliant zone near the mineralized gradient has also been identified in experimental micromechanical tests of tendon-bone samples (Rossetti et al., 2017; Deymier et al., 2017) and is believed to enhance energy absorption, allowing higher local deformation (Deymier et al., 2017). The width and the depth (i.e. drop in modulus) of the compliant region described in several computational works depend on the chosen model parameters. A dominant factor influencing the size of the compliant belt seems to be the position of the mineralization front (i.e. representing the onset of the mineralization process) within the enthesis fibrocartilage (Liu et al., 2013; Aghaei et al., 2020). Based on the current literature, values for the relative width of the compliant zone could be estimated and ranged from 13% to 59% of the total length of the insertion region, depending on the relative position of the mineralization front (starting positions from 0% to 35% of the insertion width were considered) (Genin et al., 2009; Liu et al., 2013; Aghaei et al., 2020). A fairly broad compliant attachment region (occupying a considerable width of the interface) has been deduced experimentally based on the local strain field (Fig. 4H and I) (Rossetti et al., 2017). Details of the mineral accumulation within the collagen had only a minor impact on the size of compliant region (Fig. 4C). Additionally, the drop in modulus observed in the compliant band appears to be linked to the magnitude of the angular deviation of collagen fibers (Genin et al., 2009; Liu et al., 2013; Aghaei et al., 2020). Depending on the interplay between the position of the mineralization front and the spatial variation in angular deviation, such drop may occur either in the vicinity of the tendon side (Fig. 4E) or very close to the tidemark, just before the mineralized fibrocartilage (Fig. 4C and G). The latter being in agreement with experiment findings and, most likely, corresponding to the transition from collagen type I to type II (Rossetti et al., 2017). The minimum value of the tissue elastic modulus computed in the soft attachment region was estimated to range from about 14% to 23% of the longitudinal modulus of the tendon (Genin et al., 2009; Liu et al., 2013; Aghaei et al., 2020). A higher modulus drop (i.e., a factor of 0.1 of the tendon modulus) has been assumed based on experimental measurements of the strain field at the interface (Fig. 4I) (Rossetti et al., 2017). Overall, these findings suggest that the compliant region is a remarkable construction strategy of tendon to bone attachment, which deserves additional insights in future studies. Moreover, owing to the pivotal importance of mineral and collagen arrangements for the mechanical behavior of mineralized collagen fibrils (Fratzl and Weinkamer, 2007), future modeling and computational works may still investigate mineral reinforcement aspects at the bone-soft tissue interface. For example, the staggered geometry seen in mature lamellar bone could be considered in combination with different mineral volume fractions, not only to characterize the corresponding elastic gradients but also to model deformation, failure and fracture behavior of such a transition zone.

### 2.3 Surface roughness and interlocking

**Fig. 5:**
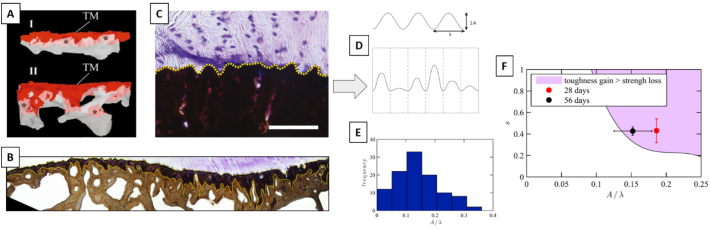
(A) Three-dimensional reconstructions (based on serial sectioning) of the interface between mineralized fibrocartilage (MFC, red) and the underlying bone (grey) in the proximal (I) and central (II) region of the Achilles tendon insertion. TM indicates the tidemark between unmineralized fibrocartilage (UFC) and MFC. (B) Roughness and interdigitations at the interface between meniscus and MFC (top) and MFC and bone (bottom). (C) Interface between mineralized (stained in black) and unmineralized (stained in purple) tissues at the supraspinatus tendon to bone insertion. The dotted line highlights the surface roughness, described in (D) as a sinusoidal wave with approximately the same wave length λ but varying amplitude A. (E) Interface waviness (defined as the ratio of A/λ) measured at the supraspinatus tendon to bone insertion in mice follows a quasi-normal distribution centered at 0.14. (F) Contour plot illustrating the region (shaded area) and the corresponding combination of roughness parameters (waviness A/λ and standard deviation s) for which the gain in toughness exceeds the loss in strength as predicted by the model in (Hu et al., 2015). Symbols represent the physiological values of the roughness parameters measured experimentally. Figures modified from (Abraham and Haut Donahue, 2013; Hu et al., 2015; Milz et al., 2002), with permission.

The compositional and microstructural gradients highlighted in the previous sections are believed to smooth the transition in material properties from tendon to bone. Nevertheless, at the sub-millimeter length scale, it is still reasonable to identify interfacial regions between the different tissues present at the enthesis and to investigate their morphology. Specifically, there are two interfaces of interests: *i*) the transition between UFC and MFC, referred to as the tidemark or mineralization front; and *ii*) the one between MFC and bone. Those regions are experimentally accessible and have been visualized using various approaches such as histology (Abraham and Haut Donahue, 2013; Hu et al., 2015; Dai et al., 2015), serial sectioning (Milz et al., 2002), quantitative backscattering electron imaging (Zizak et al., 2003; Shea et al., 2002; Ferguson et al., 2003; Gupta et al., 2005) and micro-CT (Sartori and Stark, 2020; Sartori et al., 2018). There are two main features in common to these biological interfaces: a high roughness and the presence of interlocking mechanisms, as evidenced by the analysis of different entheses including the attachment of Achilles tendon into calcaneus (Milz et al., 2002), the insertion of the supraspinatus tendon into humeral head (Hu et al., 2015), and the meniscus-tibia interface (Abraham and Haut Donahue, 2013) (Fig. 5A–C). The same is also true for the interface between mineralized articular cartilage and bone (Zizak et al., 2003; Gupta et al., 2005; Campbell et al., 2012).

Joining two dissimilar materials through a patterned interface is indeed a well-known strategy to improve the failure resistance of bimaterial attachments, and numerous works have investigated this topic considering engineering materials (Cordisco et al., 2012; Caimmi and Pavan, 2009; Hernandez et al., 2017; Kim et al., 2010; Yao and Qu, 2002). In this context, one interesting research question is about the relationship between the interface geometrical properties and the corresponding interfacial fracture toughness. A possible way to approach this problem is firstly to reduce the complexity of interface roughness by assuming an idealized profile, described for instance by a sinusoidal function (which is characterized by two parameters: amplitude A and wavelength λ). Using computational fracture mechanics tools is then feasible to relate interface geometry to fracture behavior for different mismatches in material properties between the two solids being joined. Following such route, Cordisco and colleagues (Cordisco et al., 2012) have reported that roughness increases fracture toughness with respect to a flat interface but, as a function of the material mismatch, it exists a critical threshold of A/λ above which the cracking behavior switches from single to multiple crack propagation. This transition substantially reduces the toughening effect of the interface roughness. This is an interesting finding that could be used to interpret the morphology of biological interfaces. Assuming a ratio between the elastic modulus of bone and tendon of 10, the critical value of A/λ is about 0.5 (Cordisco et al., 2012). Interestingly, roughness analysis at interface between UFC and MFC (specifically at the supraspinatus tendon to bone insertion) revealed that the aspect ratio of the interface waviness (defined as A/λ) follows an approximately normal distribution centered at 0.14 (Fig. 5D and E) (Hu et al., 2015), which is well below the 0.5 threshold. The measured surface roughness was further interpreted with an idealized 2D unit cell continuum mechanics approach (Hu et al., 2015). The model was used to calculate the strength (peak load) and the toughness (approximated by the area under the force-displacement curve) of virtual bone-tendon interfaces as a function of the interface shape. The authors considered several bimaterial unit cells with the interface approximated by a cosine wave characterized by varying amplitudes, which were assigned according to prescribed normal distributions at constant width and λ (Fig. 5D). The impact of the mean value and standard deviation of the distributions of the waviness (A/λ) on strength and toughness was estimated. Simulations showed that higher interface roughness and roughness heterogeneity both decreased the strength but increased the toughness of bimaterial attachments. There was a combination of A/λ and heterogeneity, for which the gain in toughness outweighs the loss in strength (Fig. 5F). The geometrical properties of the healthy bone-tendon interface seem to lie in that region, suggesting that not only a regular (Cordisco et al., 2012) but also a random wavy pattern can be an effective toughening mechanism at bimaterial interfaces (Hu et al., 2015). A subsequent work has analyzed the extent and shape of the bone-tendon interface at the same anatomical location (shoulder joint) but across multiple species having large differences in size (ranging from mice to dogs) (Deymier-Black et al., 2015). Interface roughness and interdigitations were well-preserved features across the considered species. In contrast to the attachment area, which showed an almost isometric scaling with muscle cross section (used as an estimation of the loading conditions), the geometrical parameters describing surface waviness (A and λ) did not change significantly with animal size. This could be explained by the fact that the increase in attachment area accompanying the increase in loading results in an almost constant interfacial stress, therefore minimizing the need to adapt interface shape (Deymier-Black et al., 2015). A second reason may be that excessively large aspect ratios may become disadvantageous for interface fracture toughness (Cordisco et al., 2012). Surface roughness is also a typical feature seen at the interface between MFC and bone. Milz and co-workers (Milz et al., 2002) analyzed the Achilles tendon insertion using serial sectioning and could reconstruct the 3D spatial morphology of the fibrocartilage-bone interface (Fig. 5A). They highlighted not only a rough surface but even the presence of a complex interlocking pattern which may be a fundamental strategy to improve attachment, for instance by providing increased resistance to shear loading. Considering the importance of surface morphology on the mechanical properties of bimaterial junctions, further modeling and computational studies should focus on this aspect. One possible research question could be the interplay between roughness and interlocking and the impact on the overall anchoring strength and toughness. Indeed, if roughness reduces attachment strength, this may be compensated by interlocking mechanisms. Additionally, a model comprising both interfaces (i.e. UFC/MFC and MFC/bone), with their specific morphologies, is still missing but could be helpful to better target the weakness of reattachment procedures, which fail to reproduce such complex interfacial shapes.

## 3 Modeling the whole bone-tendon construct: load transfer optimization and orthopedic related questions

The number of orthopedic injuries requiring the reattachment of tendons or ligaments to bone, together with the complexity of the surgical procedure and the poor long-term clinical outcome (Lu and Thomopoulos, 2013; Galatz et al., 2004), have triggered the development of computational models at the macroscopic length scale (Thomopoulos et al., 2006; Inoue et al., 2013; Wakabayashi et al., 2003; Funakoshi et al., 2008; Sano et al., 2006b; Quental et al., 2016; Mantovani et al., 2016; Liu et al., 2012). These models differ from the approaches presented in the previous sections as they essentially try to investigate the mechanical behavior of the whole bone-soft tissue complex as well as to answer specific orthopedic-related questions. The key messages highlighted by those models, together with their shortcomings, are summarized in Table 2.

**Table 2:** Modeling the whole bone-tendon construct.

Highlights
• The shape of the supraspinatus insertion is optimized to alleviate peak stresses. • Computational models illustrate the complexity of the rotator cuff with stresses heterogeneously distributed into non-trivial patterns. • The stress level at the insertion increases substantially with arm abduction and in case of damage (tears). • Reattachment procedures with transosseous sutures allow a uniform redistribution of contact pressure and reduce stress concentration.
Limitations and outlook
• Limited amount of details on geometries and material properties are used in the macroscopic and organ scale models. Future work may focus on the progressive inclusion of features highlighted in Section 2, such as material gradients and interlocking at the insertion.

### 3.1 Macroscopic models of the tendon-bone system

In comparison with the vast literature on tissue and organ level models of bones, tendons and ligaments, computational works on the entire bone-soft tissue complex including details of the enthesis are still sparse. Nevertheless, such studies are essential as they provide a picture of the stress and strain transfer from tendon to bone in the presence of selected features of the enthesis, such as the shape of the attachment area (Liu et al., 2011), the local tissue anisotropy (Thomopoulos et al., 2006) or the transition in elastic properties (Liu et al., 2012).

Bimaterial junctions are known to be at risk of highly localized stresses, especially at the edge of the junction (Liu et al., 2011; Balijepalli et al., 2016). One possible tissue level strategy to alleviate such high stresses it to optimize the shape of the attachment area. Firstly, it should be noticed that a substantial reduction of the angle between the two materials at the free edge of the attachment can be a first solution to decrease the peak stresses developed at the interface (Liu et al., 2011). This may be a biomechanical motivation of the splay-out of collagen fibers observed experimentally at the bone-tendon interface (Fig. 3A–B) (Rossetti et al., 2017). Peak stresses can also be reduced by optimizing the gross morphology of the attachment area. Using an idealized continuum FE model in combination with a gradient-based shape optimization algorithm, it was shown that the shape of the bone-tendon insertion region can be optimized to practically eliminate peak stresses caused by mismatches in material properties. Besides, the optimized shape differed when comparing pristine entheses with reattached tendons through scar tissue (Liu et al., 2011).

**Fig. 6:**
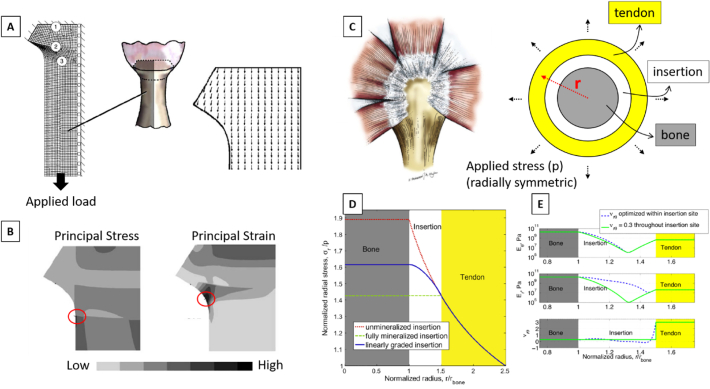
(A) Idealized FE model of the tendon-bone complex based on measured geometrical features at the rat shoulder. The numbers indicate: (1) the bone-mineralized cartilage interface, (2) the tidemark between mineralized fibrocartilage (MFC) and unmineralized fibrocartilage (UFC) and (3) the tendon. The right image shows one of the simulated scenarios for the local predominant orientation of collagen fibers, following the outward splay of interface region. (B) Simulated principal stress and strain. Peak values are localized beneath the outward splay of the tendon (as highlighted by the red circles). (C) Anatomical side view (left) and idealized model (right) of the bone-tendon system at the rotator cuff, showing the rotator cuff tendons wrapping around the humeral head. The axisymmetric idealized model comprises the bone, the bone-tendon interface and the tendon. Material properties of bone and tendon were fixed, whereas properties of the interface were allowed varying. The applied radial stress simulated muscle loading. (D) Variation of normalized radial stress (along the radial direction) for unmineralized (tendon like), fully mineralized (bone like), and linearly graded interface region. In all cases, stresses are substantially higher than the applied stress. This happens not only at the interface but also within the bone and the tendon. (E) Distribution of material properties (i.e., radial elastic modulus, tangential elastic modulus and Poisson ratio of the insertion) resulting from the minimization of radial stress. A compliant region (with stiffness lower than tendon and bone) appears at the interface. Allowing Poisson ratio to vary across the insertion has an effect only on the tangential modulus. Figures modified from (Thomopoulos et al., 2006; Liu et al., 2012), with permission.

The gross shape of the insertion site at the millimeter level is undoubtedly an important parameter for the efficiency of the enthesis. A computational model combining the morphology of the attachment area with orientation-dependent mechanical behavior of the enthesis was developed by Thomopoulos and coworkers (Thomopoulos et al., 2006). The authors modeled the supraspinatus tendon to bone complex using a 2D idealized continuum FE approach which captured, to some extent, the observed outward splay geometry of rat supraspinatus tendon insertion and accounted for the elastic anisotropy caused by locally different orientation of tendon fibers (Fig. 6A). Various scenarios were considered for the distribution of collagen orientation, including a random, a mechanically optimized and an experimentally based arrangement. The FE results, analyzed in terms of stress and strain concentrations (Fig. 6B), underlined that the spatial distribution of material anisotropy had a very large impact on the overall stress and strain transfer between tendon and bone. Furthermore, the physiological arrangement of collagen fibers at the enthesis seems to be dictated by multiple requirements such as reducing peak stresses, shielding the insertion splayed geometry and, at the same time, maximizing the stiffness along the main pulling direction of the tendon.

As seen in Section 2.1, computational models of partially mineralized collagen fibers predict a decrease in elastic modulus at the insertion site to values lower than tendon and bone (Genin et al., 2009; Liu et al., 2012). To better understand the possible biomechanical reasons of such counterintuitive behavior, a tissue-scale biomechanical model was developed, describing essential geometrical features and loading conditions of the bone-tendon complex at the shoulder joint (Liu et al., 2012). The model features three axisymmetric concentric cylinders, with an isotropic bone core encompassed by an orthotropic cylindrical tendon and with the bone-tendon interface described as a ring in between (Fig. 6C). Different material properties were assigned to the ring-like interface region, including unmineralized (like tendon), fully mineralized (like bone) and linearly graded transition from tendon to bone. The model was solved using a continuum structural mechanics approach and showed that, in all the considered scenarios, the radial stress increased and was from 40% to 90% higher than the applied external stress (Fig. 6D). Conversely, if local material properties within the interface region were allowed varying according to a numerical optimization scheme minimizing radial stress, the obtained distribution of elastic modulus showed a minimum value below that of tendon and bone (Fig. 6E). However, this happened at the expense of tangential stresses which increased to values above the applied stress. A similar approach was then used to demonstrate that the level of peak stresses at the shoulder joint is a much conserved feature across multiple species, despite the large variations in key anatomical characteristics of the enthesis (Saadat et al., 2016). Those results illustrate that interface anisotropy may be advantageous when connecting an orthotropic material to an isotropic one. Interestingly, such anisotropy can be caused by different factors, and this is a topic worth of comparison among the different modeling approaches, as anisotropy can have a large impact on stress concentration at bimaterial interfaces. For example, considering modeling approaches described in Section 2.2, anisotropy can result from an intrinsic behavior of the basic constituents of the model (e.g., collagen fibers idealized as transversally isotropic elements) (Liu et al., 2013) or it can as well emerge from the shape and spatial arrangement of basic isotropic building blocks (Aghaei et al., 2020; Saadat et al., 2015).

### 3.2 Organ-scale models targeting orthopedic questions

Musculoskeletal injuries involving enthesis are a common clinical problem with an increased incidence in the aged population (Yamaguchi et al., 2006), and with related treatment strategies suffering from complications and, in some cases, high failure rates (Galatz et al., 2004). The most frequently injured locations include the knee and the shoulder joints. At the knee joint, ACL rupture often requires the replacement of the damaged ligament with biological or synthetic grafts (Heming et al., 2007). Despite the fact that the surgical procedure is well-established, about 50% of the patients still experience pain one year post surgery (Keene, 2000) and have an increased probability (up to 50%) of suffering from osteoarthritis in the following years (Simon et al., 2015). At the shoulder joint, the rotator cuff tear is a major cause of pain and instability (Gombera and Sekiya, 2014). The rotator cuff is a fairly complex joint featuring several muscles and tendons (Fig. 6). Due to the specific loading conditions and anatomical position, the supraspinatus tendon is the most frequently damaged soft tissue of the shoulder (Martin et al., 1998; Lake et al., 2009; Palastanga et al., 2006), being susceptible to partial or full-thickness tears (Andarawis-Puri et al., 2009; Engelhardt et al., 2016; Thunes et al., 2015). Massive rotator cuff injuries may necessitate the reattachment of the tendon to the bony insertion with sutures: different surgical options are available (Mantovani et al., 2016) but, contrary to ACL repair, there is no consensus on a gold-standard management approach (Quental et al., 2016; Mantovani et al., 2016). Reattachment procedures are exposed to high risk of failure, with re-injury rates as high as 94% (Galatz et al., 2004; D Harryman et al., 1991). Moreover, effects of unloading (e.g., due to disuse or paralysis) at the rotator cuff have been experimentally investigated (Deymier et al., 2019) and revealed alterations across multiple length scales that could lead to increased risk of injury. For those reasons, numerous clinical and biomechanical studies have focused on both healthy and damaged supraspinatus-humerus complex and, therefore, we will briefly review some computational models at this anatomical location, which have been developed with the overall goal of understanding the main biomechanical reasons for damage and failure as well as to improve treatment options (Inoue et al., 2013; Wakabayashi et al., 2003; Funakoshi et al., 2008; Sano et al., 2006b; Quental et al., 2016; Mantovani et al., 2016).

#### 3.2.1 Simulations of healthy bone-tendon complex

**Fig. 7:**
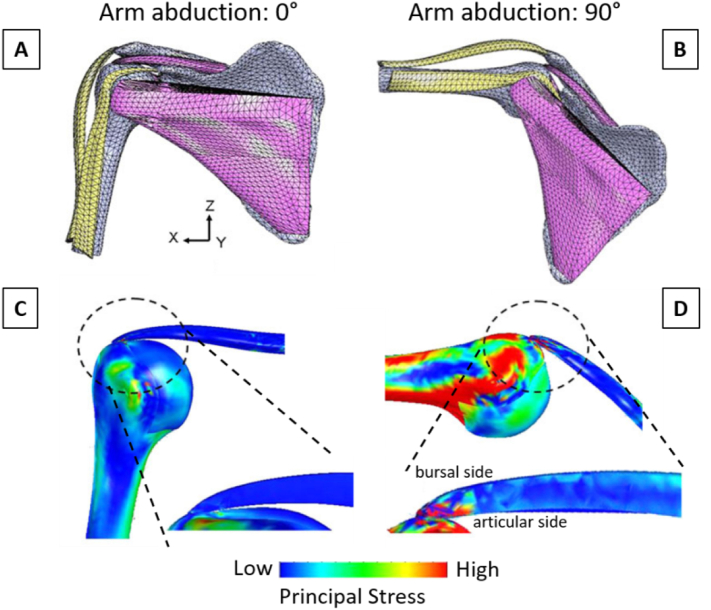
Three-dimensional FE model of a normal shoulder joint at (A) 0° and (B) 90° arm abduction. Corresponding surface distributions of maximum principal stress are shown in (C) and (D). The inserts show a sagittal cut through the anterior section and highlight the increase in stress caused by a lifted arm, especially at the articular side of the tendon, which might explain the observed high tear incidence at that location. Figures modified from (Inoue et al., 2013), with permission.

One important research question which has been investigated with computational models of healthy bone-tendon complex is about the position and the magnitude of peak stresses during arm abduction (Inoue et al., 2013; Wakabayashi et al., 2003). Using a fairly simple 2D approach with geometrical information based on magnetic resonance images, Wakabayashi and co-workers (Wakabayashi et al., 2003) developed a multi-tissue model, with the tendon fully bonded to the humeral head and with the presence of (frictionless) contact between tendon and bone , and rather simplified material properties. Starting from the arm at resting position (0° abduction), peak stresses were located at the contact region between tendon and bone, which happened away from the insertion location. Increasing the abduction angle, the highest stressed region moved towards the enthesis and reached it at about 60° abduction. Such behavior was confirmed in a subsequent refined 3D model, based on computed tomography (CT) scans and including more anatomical details, such as the three major rotator cuff tendons and the middle fibers of the deltoid muscle (Fig. 7A–B) (Inoue et al., 2013). The authors also incorporated additional complexity in material definition, all soft tissues being modeled with non-linear material properties, and the possible interaction between bone and tendon surfaces during abduction was described by frictionless contact. The model confirmed a substantial increase of peak stresses with abduction, in particular closer to the insertion of the supraspinatus at anterior edge of the articular side (Fig. 7C–D). The results interestingly hinted for the presence of shear stresses caused by differences in the stress state across the tendon from the articular (highly loaded in tension) to the bursal (less loaded in tension) surface. Shear load in tendon might cause delamination, which could explain the partial-thickness tears often observed in that location (Gwak et al., 2015; Iwashita et al., 2018).

#### 3.2.2 Simulations of damaged bone-tendon complex and reattachment procedures

**Fig. 8:**
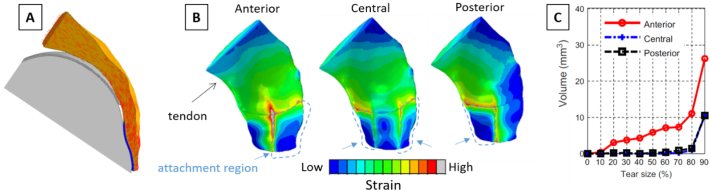
(A) Two-dimensional cross-sectional view of the computational model for the tendon-humerus complex featuring the humerus (light grey), the cartilage (grey), the fibrocartilage (blue), and tendon (orange). The predominant direction of selected tendon fibers is represented by red lines. (B) Illustration of the maximum principal strains computed for a 200 N load in a 50% damaged tendon-humerus complex with full-thickness tears on anterior, central or posterior location. (E) Corresponding volume of tendon tissue with strain above the failure threshold (estimated to be at 24.5%) as a function of tear size. Figures modified from (Quental et al., 2016), with permission.

Computational models offer the possibility to elucidate the impact of damage (in the form of tears) on the overall mechanical competence of tendon-bone constructs. One clear advantage of virtual models is that different degrees of damage can be inserted in a controlled manner at selected locations and the detrimental role can be estimated (Sano et al., 2006b; Quental et al., 2016). Sano and co-workers, for example, simulated partial-thickness tears on three different anatomical positions close to the tendon insertion (i.e., articular surface, bursal surface and tendon midsubstance) using a simplified 2D model of the supraspinatus-humerus complex (Sano et al., 2006b). Interestingly, inserting damage not only caused stress concentration around the damaged region, but also increased the level of stress at the bony insertion. Furthermore, the biomechanical impact of damage locations depended on arm position: at 0° abduction, damage on the bursal side was the worst-case scenario, whereas at 60° abduction, tears at the articular surface induced the highest stress. A subsequent and extensive 3D study was conducted by Quental and co-workers (Quental et al., 2016), with virtual samples of the supraspinatus-humerus complex based on the Visible Human Project dataset (Spitzer et al., 1996). The model included not only bone and tendon but also articular and enthesis fibrocartilage (Fig. 8A), assumed to behave like hyperelastic Neo-Hookean materials. Tears of increasing severity were introduced right at bone-tendon attachment by decreasing the insertion area at different locations (i.e., anterior, central and posterior). The authors evaluated the impact of tears by monitoring the distributions of principal strains (Fig. 8B), as this quantity seems to correlate with damage propagation, at least in vitro (Andarawis-Puri et al., 2009). Results indicated that tears located on the anterior side had a greater risk of propagation, as the volume of tendon tissue exceeding a typical tissue failure strain (assumed at 24.5% strain) was the highest (Fig. 8C). Overall this study represents a good example of how computational models can support and help the biomechanical interpretation of orthopedic observations.

**Fig. 9:**
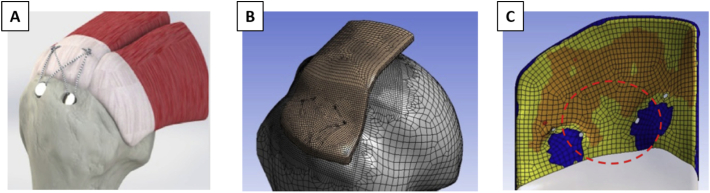
(A) Solid model for the FE analysis of the transosseous equivalent technique. (B) 3D mesh used in the simulations. (C) Qualitative maps of the supraspinatus-bone contact layout; orange represents the area in contact with a positive applied pressure. The free surfaces are in yellow, and the absence of any contact (device insertion areas) is shown in blue. Figures modified from (Mantovani et al., 2016), with permission.

Computational models have also been developed to investigate the impact of surgical approaches reattaching soft tissues to bone (Funakoshi et al., 2008; Mantovani et al., 2016). Funokashi and co-workers performed an early study to compare different orthopedic reattachment strategies at the rotator-cuff humerus enthesis including a traditional double row fixation (Huijsmans et al., 2007) and a surface holding repair technique either with transosseous sutures or with knotless anchors (Funakoshi et al., 2008). The authors performed experimental in vitro tests on bovine shoulders and developed simplified FE models to support the interpretation of the experimental findings, by characterizing the link between the reattachment options and the corresponding stress distribution in the tendon-bone complex. The surface-holding repair with transosseous sutures provided the most rigid fixation and also prevented the high stress concentration seen with the double-row repair. This could explain the improved failure resistance of such reattachment strategy observed in the experiments. A subsequent computational work confirmed the biomechanical advantages of transosseous sutures (Fig. 9A) (Mantovani et al., 2016). The authors simulated three different repair approaches (i.e., single row, double row and transosseous equivalent) now using a 3D model with a patient-specific geometry based on a CT scan (Fig. 9B). FE results showed that the transosseous equivalent suture lead to an increase in contact area and a more uniform distribution of contact pressure between tendon and bone (Fig. 9C), which are two factors linked with the effectiveness of rotator cuff repair (Tuoheti et al., 2005).

Future continuum FE-based models targeting reattachment procedures could be enriched by including a more detailed description of entheses microstructure and biomechanical behavior as presented in Section 2. Clearly, it is highly challenging to bridge such a gap between the organ scale (relevant to address applied orthopedic questions) and the (sub-)micrometer level (suited to investigate basic anchoring strategies). To some extent, computational multiscale models could be helpful and may, for example, trigger the development of novel (biomimetic) adhesive (Avgoulas et al., 2019), recapitulating for certain aspects the material behavior of healthy entheses.

## 4 Limitations and outlook

Entheses are essential players of the musculoskeletal system, which have the challenging task of attaching dissimilar tissues over a very tiny region. In this review, we have presented some key features allowing entheses to facilitate load transmission from soft tissues to bone (summarized in Table 3). In particular, we have highlighted the valuable contributions of modeling and computer simulations for the investigation of attachment strategies. Models have provided insights into the structure-function relationship at entheses, which would not have been possible using experiments only. In general, models have the considerable advantage to be able to simulate scenarios that would be either impossible or costly to reproduce experimentally, such as assessing the mechanical impact of an individual feature (e.g., fiber alignment) or a gradual and controlled variation in a selected parameter (e.g., tendon tear size). Considering Section 2 (reviewing the link between enthesis microstructure and composition to mechanical properties), one modeling approach still not much explored for the enthesis could be based on discrete network models, accounting for individual fibers (or fiber bundles) attaching to bone at discrete anchoring points. Although computationally expensive, such modeling route may reveal novel biomechanical aspects which are not captured when describing the enthesis as a continuum material. In analogy, discrete computational models of trabecular bone accounting for individual trabeculae have boosted our understating on deformation, failure and even mechanical adaptation of trabecular bone, by detailing mechanisms not easily evident with continuum modeling (Wang et al., 2015; Ruffoni et al., 2012; Maurer et al., 2015; Dunlop et al., 2009; Schulte et al., 2013). Furthermore, future models should target not only the local heterogeneous stress distribution but also failure aspects such as damage initiation and propagation. This could provide a broader picture on enthesis resilience and robustness to various loading conditions. Indeed, entheses may be designed by nature not only to minimize possible stress concentrations due to material mismatches, but also to cope with the unavoidable presence of damage. Similarly to bone (Razi et al., 2020) and many other biological materials (Fratzl and Weinkamer, 2007; Huang et al., 2019), entheses may feature multiple toughening mechanisms at different length scales to hamper damage propagation. The presence of a compliant region with stiffness lower than tendon and bone is most likely one of such toughening strategies, deserving extra attention in future damage-based computational efforts. This is also a remarkable construction principle that may trigger the development of novel bioinspired adhesive to join strongly dissimilar materials. Finely tuned spatial variations in elastic modulus is only one aspect of the complex transition in material properties when going from tendon to bone, as the two tissues feature also different failure strain, fracture toughness, as well as viscoelastic behavior. Indeed, other material-level properties may also display a somewhat gradual transition across the enthesis, which need to be characterized by local experimental methods. Once known, these material parameters may then be cast into computational models going beyond linear elasticity to obtain a more realistic picture of the load transfer mechanism at the enthesis.

**Table 3:** Computational and experimental findings regarding attachment strategies, load optimization mechanisms and overall biomechanical competence of tendon (and ligament) to bone junctions.

Investigated feature	Paper	Studied enthesis & species	Approach of the study	Main findings
2.1 *Fiber architecture*	(Genin et al., 2009)	Shoulder joint (rat supraspinatus tendon)	Continuum mechanics 3D unit cell model of a hypothetical enthesis without mineral	Angular deviation of fibers alone causes a drop of elastic modulus.
	(Rossetti et al., 2017)	Ankle joint (pig Achilles tendon)	Experimental analysis combined with displacement field mapping	Tendon fibers are splaying and unravelling to anchor to bone. There is an angle-dependent force redistribution among fibers.
	(Sartori and Stark, 2020)	Ankle joint (mice Achilles tendon)	Experimental analysis combined with fiber tracking algorithm	Details aspects of fiber curving, branching and twisting before entering bone.
2.2 *Mineral reinforcement*	(Wopenka et al., 2008)	Shoulder joint (rat supraspinatus tendon)	Experimental assessment of mineral content across insertion	The variation of mineral content across the interface is fairly linear and occurs over a region of ~120 μm.
	(Genin et al., 2009)	Shoulder joint (rat supraspinatus tendon)	Continuum mechanics 3D unit cell model with different modes of mineralization	The resulting variation in tissue stiffness across insertion is highlighting a compliant region with a stiffness lower than tendon and bone.
	(Liu et al., 2013)	Idealized models based on data from shoulder joint (rat supraspinatus tendon)	Micromechanical 3D model of collagen stiffening based on nanoscale mineralization details (initiation and sequence)	Details of the mineralization process can have a large impact on tissue stiffening, notably on the spatial gradient rather than on the compliant region.
	(Aghaei et al., 2020)	Idealized models based on generalized entheses data and data from shoulder joint (rat supraspinatus tendon)	Micromechanical continuum 3D model based on homogenization steps including nano-, sub-micro- and microstructural information	The width and modulus drop of the compliant region vary with the mineralization front position and the magnitude of the fibers angular deviation, respectively.
2.3 *Surface roughness and interlocking*	(Cordisco et al., 2012)	General approach	Fracture mechanics tools applied on an idealized sinusoidal interface	Roughness increases toughness but there is a threshold above which multiple cracks start forming hence reducing toughness.
	(Hu et al., 2015)	Shoulder joint (rat supraspinatus tendon)	Continuum mechanics 2D idealized bimaterial unit cell model	Geometrical properties of the interface lay in a region where the gain in toughness outweighs the loss in strength.
	(Milz et al., 2002)	Ankle joint (human Achilles tendon)	Experimental analysis combined with 3D reconstruction of morphology	In addition to roughness, there is a complex interlocking pattern at the interface.
3.1 *Load transfer optimization*	(Liu et al., 2011)	Shoulder joint (human supraspinatus tendon)	Continuum 2D FE model combined with a shape optimization algorithm	Shape of the insertion can be optimized to eliminate peak stresses.
	(Thomopoulos et al., 2006)	Shoulder joint (rat supraspinatus tendon)	Continuum 2D FE model including local anisotropy and experimentally observed outward splay geometry.	Experimentally based arrangement reduces peak stress and shields the insertion splay while maximizing the stiffness.
	(Liu et al., 2012)	Shoulder joint (human rotator cuff tendons)	Continuum 2D structural model combined with numerical optimization (minimizing radial stress)	Resulting elastic modulus of the interface has a minimum lower than tendon and bone (at the expense of tangential stress).
3.2 *Peak stress regions* (*and link with tear*)	(Inoue et al., 2013)	Shoulder joint (human supraspinatus tendon)	2D simplified FE model with linear material properties	Peak stress move towards insertion with arm abduction.
	(Wakabayashi et al., 2003)	Shoulder joint (human supraspinatus tendon)	3D FE model comprising non-linear material properties	Differences in stress state between both sides of the tendon with arm abduction can cause shear stress and therefore delamination tears.
3.2 *Damage and reattachment procedures*	(Sano et al., 2006b)	Shoulder joint (human supraspinatus tendon)	2D simplified FE model with partial thickness tears	Damage cause stress concentration around tears but also increase stress at the bony insertion. Stress state depends on arm abduction.
	(Quental et al., 2016)	Shoulder joint (human supraspinatus tendon)	3D FE model including fibrocartilage modeling, with increasing size tears	Tears on the anterior side have a greater risk of propagation.
	(Funakoshi et al., 2008)	Shoulder joint (bovine supraspinatus tendon)	2D FE model with three types of reattachment procedures	Surface holding repair with transosseous sutures prevents high stress concentration.
	(Mantovani et al., 2016)	Shoulder joint (human supraspinatus tendon)	3D FE model with three types of reattachment procedures	Transosseus sutures lead to increased contact area and more uniform distribution of contact pressure.

Computer simulations at the organ scale have allowed answering orthopedic relevant questions, with many studies focusing on the rotator cuff, as reviewed in Section 3. In general, organ scale models lack a detailed description of the enthesis, probably due to the high computational cost needed to include micrometer level information on enthesis structure and material behavior. Depending on the specific research questions investigated with the model, a realistic representation of the enthesis may or may not be necessary. However, selecting and quantifying which aspects of the enthesis (e.g. spatial gradients, interlocking structure, compliant zone) should be included into organ level models may help to increase the ability of those studies to predict surgical outcomes.

From a mechanobiological viewpoint, musculoskeletal tissues have the remarkable ability to modify their structure in response to functional mechanical demands. Bone and tendon mechanobiology are well-established fields, which have seen important contributions coming from modeling and simulations. For instance, the knowledge of the local stress and strain at the tissue level obtained thanks to image-based microstructural FE analysis, combined with experimental information on bone remodeling, has allowed unravelling fundamental aspects on the mechanical control of bone regeneration (Schulte et al., 2013; Christen et al., 2014; Li et al., 2019; Razi et al., 2015). Enthesis mechanobiology is still a growing field and future in silico approaches should be used to compute the local mechanical environment at the cellular level, for instance during various stages of enthesis development, to elucidate the mechanical cues underlying the formation and the maintain of such a complex biological interface. Knowing the local mechanical environment within enthesis fibrocartilage and around fibrochondrocytes may also improve the understanding of the biomechanical factors contributing to enthesis degeneration.

## Declaration of competing interest

The authors have no conflict of interest.

## References

[bb0005] Abraham A.C., Haut Donahue T.L. (2013). From meniscus to bone: a quantitative evaluation of structure and function of the human meniscal attachments. Acta Biomater..

[bb0010] Aghaei A. (2020). Assessing the effective elastic properties of the tendon-to-bone insertion: a multiscale modeling approach. Biomech. Model. Mechanobiol..

[bb0015] Andarawis-Puri N., Ricchetti E.T., Soslowsky L.J. (2009). Rotator cuff tendon strain correlates with tear propagation. J. Biomech..

[bb0020] Apostolakos J. (2014). The enthesis: a review of the tendon-to-bone insertion. Muscles, ligaments and tendons journal.

[bb0025] Armitage O.E., Oyen M.L. (2017). Indentation across interfaces between stiff and compliant tissues. Acta Biomater..

[bb0030] Avgoulas E.I. (2019). Adhesive-based tendon-to-bone repair: failure modelling and materials selection. J. R. Soc. Interface.

[bb0035] Balijepalli R.G. (2016). Numerical simulation of the edge stress singularity and the adhesion strength for compliant mushroom fibrils adhered to rigid substrates. Int. J. Solids Struct..

[bb0040] Ban E. (2019). Strong triaxial coupling and anomalous Poisson effect in collagen networks. Proc. Natl. Acad. Sci..

[bb0045] Bannerman A., Paxton J.Z., Grover L.M. (2014). Imaging the hard/soft tissue interface. Biotechnol. Lett..

[bb0050] Bedi A. (2010). Effect of early and delayed mechanical loading on tendon-to-bone healing after anterior cruciate ligament reconstruction. The Journal of Bone and Joint Surgery. American volume.

[bb0055] Benjamin M., Ralphs J.R. (1998). Fibrocartilage in tendons and ligaments -an adaptation to compressive load. J. Anat..

[bb0060] Benjamin M. (2002). The skeletal attachment of tendons—tendon ‘entheses’. Comp. Biochem. Physiol. A Mol. Integr. Physiol..

[bb0065] Benjamin M. (2006). Where tendons and ligaments meet bone: attachment sites (’entheses’) in relation to exercise and/or mechanical load. J. Anat..

[bb0070] Bi X. (2005). A novel method for determination of collagen orientation in cartilage by Fourier transform infrared imaging spectroscopy (FT-IRIS). Osteoarthr. Cartil..

[bb0075] Biewener A.A., Fratzl P. (2008). Tendons and ligaments: structure, mechanical behavior and biological function. Collagen: Structure and Mechanics.

[bb0080] Bonnevie E.D., Mauck R.L. (2018). Physiology and engineering of the graded interfaces of musculoskeletal junctions. Annu. Rev. Biomed. Eng..

[bb0085] Boote C., Dennis S., Meek K. (2004). Spatial mapping of collagen fibril organisation in primate cornea—an X-ray diffraction investigation. J. Struct. Biol..

[bb0090] Borah B. (2006). Long-term risedronate treatment normalizes mineralization and continues to preserve trabecular architecture: sequential triple biopsy studies with micro-computed tomography. Bone.

[bb0095] Bounoure L. (2014). The role of the renal ammonia transporter rhcg in metabolic responses to dietary protein. J. Am. Soc. Nephrol..

[bb0100] Boys A.J. (2017). Next generation tissue engineering of orthopedic soft tissue-to-bone interfaces. MRS Communications.

[bb0105] Boys A.J. (2019). Understanding the stiff-to-compliant transition of the meniscal attachments by spatial correlation of composition, structure, and mechanics. ACS Appl. Mater. Interfaces.

[bb0110] Bromage T.G. (2003). Circularly polarized light standards for investigations of collagen fiber orientation in bone. The Anatomical Record Part B: The New Anatomist.

[bb0115] Buenzli P.R., Sims N.A. (2015). Quantifying the osteocyte network in the human skeleton. Bone.

[bb0120] Caimmi F., Pavan A. (2009). An experimental evaluation of glass–polymer interfacial toughness. Eng. Fract. Mech..

[bb0125] Calejo I., Costa-Almeida R., Gomes M.E., Turksen K. (2019). Cellular complexity at the interface: challenges in enthesis tissue engineering. Cell Biology and Translational Medicine, Volume 5: Stem Cells: Translational Science to Therapy.

[bb0130] Campbell S.E., Ferguson V.L., Hurley D.C. (2012). Nanomechanical mapping of the osteochondral interface with contact resonance force microscopy and nanoindentation. Acta Biomater..

[bb0135] Christen P. (2014). Bone remodelling in humans is load-driven but not lazy. Nat. Commun..

[bb0140] Cordisco F.A. (2012). Toughness of a patterned interface between two elastically dissimilar solids. Eng. Fract. Mech..

[bb0145] Currey J.D. (2002). Bones: Structure and Mechanics.

[bb0150] D Harryman L.M., Wang K., Jackins S., Richardson M., Matsen F. (1991). Repairs of the rotator cuff. Correlation of functional results with integrity of the cuff. The Journal of Bone & Joint Surgery.

[bb0155] Dai C. (2015). Regional fibrocartilage variations in human anterior cruciate ligament tibial insertion: a histological three-dimensional reconstruction. Connect. Tissue Res..

[bb0160] Daxer A., Fratzl P. (1997). Collagen fibril orientation in the human corneal stroma and its implication in keratoconus. Invest. Ophthalmol. Vis. Sci..

[bb0165] De Falco P. (2017). Staggered fibrils and damageable interfaces lead concurrently and independently to hysteretic energy absorption and inhomogeneous strain fields in cyclically loaded antler bone. ACS Biomaterials Science & Engineering.

[bb0170] Derwin K.A. (2018). Enthesis repair: challenges and opportunities for effective tendon-to-bone healing. JBJS.

[bb0175] Desmorat R., Leckie F.A. (1998). Singularities in bi-materials: parametric study of an isotropic/anisotropic joint. European Journal of Mechanics - A/Solids.

[bb0180] Deymier A.C. (2017). Micro-mechanical properties of the tendon-to-bone attachment. Acta Biomater..

[bb0185] Deymier A.C. (2019). The multiscale structural and mechanical effects of mouse supraspinatus muscle unloading on the mature enthesis. Acta Biomater..

[bb0190] Deymier-Black A.C. (2015). Allometry of the tendon enthesis: mechanisms of load transfer between tendon and bone. J. Biomech. Eng..

[bb0195] Dong S. (2017). A three-dimensional collagen-fiber network model of the extracellular matrix for the simulation of the mechanical behaviors and micro structures. Computer Methods in Biomechanics and Biomedical Engineering.

[bb0200] Dunlop J. (2009). New suggestions for the mechanical control of bone remodeling. Calcif. Tissue Int..

[bb0205] Dunlop J.W.C., Weinkamer R., Fratzl P. (2011). Artful interfaces within biological materials. Mater. Today.

[bb0210] Engelhardt C. (2016). Effect of partial-thickness tear on loading capacities of the supraspinatus tendon: a finite element analysis. Computer Methods in Biomechanics and Biomedical Engineering.

[bb0215] Fan K. (2015). Effects of toughness mismatch on failure behavior of Bi-material interfaces. Procedia Engineering.

[bb0220] Felsenthal N., Zelzer E. (2017). Mechanical regulation of musculoskeletal system development. Development.

[bb0225] Ferguson V.L., Bushby A.J., Boyde A. (2003). Nanomechanical properties and mineral concentration in articular calcified cartilage and subchondral bone. J. Anat..

[bb0230] Fratzl P., Fratzl P. (2008). Collagen: Structure and Mechanics, an Introduction, in Collagen: Structure and Mechanics.

[bb0235] Fratzl P., Weinkamer R. (2007). Nature’s hierarchical materials. Prog. Mater. Sci..

[bb0240] Funakoshi T. (2008). In vitro and finite element analysis of a novel rotator cuff fixation technique. J. Shoulder Elb. Surg..

[bb0245] Galatz L.M. (2004). The outcome and repair integrity of completely arthroscopically repaired large and massive rotator cuff tears. Journal of Bone and Joint Surgery-American Volume.

[bb0250] Galvis L. (2013). Polarized Raman anisotropic response of collagen in tendon: towards 3D orientation mapping of collagen in tissues. PLoS One.

[bb0255] Gao H. (2003). Materials become insensitive to flaws at nanoscale: lessons from nature. Proc. Natl. Acad. Sci. U. S. A..

[bb0260] Gasser T.C., Ogden R.W., Holzapfel G.A. (2006). Hyperelastic modelling of arterial layers with distributed collagen fibre orientations. J. R. Soc. Interface.

[bb0265] Genin G.M. (2009). Functional grading of mineral and collagen in the attachment of tendon to bone. Biophys. J..

[bb0270] Georgiadis M., Müller R., Schneider P. (2016). Techniques to assess bone ultrastructure organization: orientation and arrangement of mineralized collagen fibrils. J. R. Soc. Interface.

[bb0275] Gianotti S.M. (2009). Incidence of anterior cruciate ligament injury and other knee ligament injuries: a national population-based study. J. Sci. Med. Sport.

[bb0280] Gombera M.M., Sekiya J.K. (2014). Rotator cuff tear and glenohumeral instability: a systematic review. Clin. Orthop. Relat. Res..

[bb0285] Gupta H.S. (2005). Two different correlations between nanoindentation modulus and mineral content in the bone-cartilage interface. J. Struct. Biol..

[bb0290] Gupta H.S. (2006). Cooperative deformation of mineral and collagen in bone at the nanoscale. Proc. Natl. Acad. Sci. U. S. A..

[bb0295] Gwak H.-C. (2015). Delaminated rotator cuff tear: extension of delamination and cuff integrity after arthroscopic rotator cuff repair. J. Shoulder Elb. Surg..

[bb0300] Harrington M.J., Waite J.H. (2009). How nature modulates a fiber’s mechanical properties: mechanically distinct fibers drawn from natural mesogenic block copolymer variants. Adv. Mater..

[bb0305] Hauch K.N. (2009). Nanoindentation of the insertional zones of human meniscal attachments into underlying bone. J. Mech. Behav. Biomed. Mater..

[bb0310] Heming J.F., Rand J., Steiner M.E. (2007). Anatomical limitations of transtibial drilling in anterior cruciate ligament reconstruction. Am. J. Sports Med..

[bb0315] Hernandez E. (2017). Toughness amplification in copper/epoxy joints through pulsed laser micro-machined interface heterogeneities. Sci. Rep..

[bb0320] Hu Y.Z. (2015). Stochastic interdigitation as a toughening mechanism at the interface between tendon and bone. Biophys. J..

[bb0325] Huang W. (2019). Multiscale toughening mechanisms in biological materials and bioinspired designs. Adv. Mater..

[bb0330] Huijsmans P.E. (2007). Arthroscopic rotator cuff repair with double-row fixation. JBJS.

[bb0335] Hull, D. and T.W. Clyne, An Introduction to Composite Materials. 2 ed. Cambridge Solid State Science Series. 1996, Cambridge: Cambridge University Press.

[bb0340] Inoue A. (2013). Nonlinear stress analysis of the supraspinatus tendon using three-dimensional finite element analysis. Knee Surg. Sports Traumatol. Arthrosc..

[bb0345] Iwashita S. (2018). Characteristics of the patients with delaminated rotator cuff tear. SICOT-J.

[bb0350] Jäger I., Fratzl P. (2000). Mineralized collagen fibrils: a mechanical model with a staggered arrangement of mineral particles. Biophys. J..

[bb0355] Ji B., Gao H. (2004). Mechanical properties of nanostructure of biological materials. Journal of the Mechanics and Physics of Solids.

[bb0360] Kazanci M. (2006). Bone osteonal tissues by Raman spectral mapping: orientation–composition. J. Struct. Biol..

[bb0365] Keene G. (2000). Arthroscopic reconstruction of the anterior cruciate ligament. A comparison of patellar tendon autograft and four-strand hamstring tendon autograft. The American journal of sports medicine.

[bb0370] Kim W.-S. (2010). Evaluation of mechanical interlock effect on adhesion strength of polymer–metal interfaces using micro-patterned surface topography. Int. J. Adhes. Adhes..

[bb0375] Lake S.P. (2009). Effect of fiber distribution and realignment on the nonlinear and inhomogeneous mechanical properties of human supraspinatus tendon under longitudinal tensile loading. Journal of orthopaedic research: official publication of the Orthopaedic Research Society.

[bb0380] Lee B. (2014). A three-dimensional computational model of collagen network mechanics. PLoS One.

[bb0385] Lee N., Robinson J., Lu H. (2016). Biomimetic strategies for engineering composite tissues. Curr. Opin. Biotechnol..

[bb0390] Lerebours C. (2020). Mineral density differences between femoral cortical bone and trabecular bone are not explained by turnover rate alone. bioRxiv.

[bb0395] Li Z. (2019). Mechanical regulation of bone formation and resorption around implants in a mouse model of osteopenic bone. J. R. Soc. Interface.

[bb0400] Liu Y.X. (2011). Mechanisms of bimaterial attachment at the interface of tendon to bone. Journal of Engineering Materials and Technology-Transactions of the Asme.

[bb0405] Liu Y.X. (2012). Bi-material attachment through a compliant interfacial system at the tendon-to-bone insertion site. Mech. Mater..

[bb0410] Liu Y. (2013). Modelling the Mechanics of Partially Mineralized Collagen Fibrils, Fibres and Tissue.

[bb0415] Locke R.C., Abraham A.C., Killian M.L. (2017). Orthopedic interface repair strategies based on native structural and mechanical features of the multiscale enthesis. ACS Biomaterials Science & Engineering.

[bb0420] Lu H.H., Thomopoulos S. (2013). Functional attachment of soft tissues to bone: development, healing, and tissue engineering. Annu. Rev. Biomed. Eng..

[bb0425] Lukas C. (2011). The heterogeneous mineral content of bone-using stochastic arguments and simulations to overcome experimental limitations. J. Stat. Phys..

[bb0430] Lukas C. (2011). Quantification of the interplay between mineralization and remodeling in trabecular bone assessed by in vivo micro-computed tomography. Bone.

[bb0435] Lyman S. (2009). Epidemiology of anterior cruciate ligament reconstruction: trends, readmissions, and subsequent knee surgery. JBJS.

[bb0440] Mantovani M. (2016). A 3D finite element model for geometrical and mechanical comparison of different supraspinatus repair techniques. J. Shoulder Elb. Surg..

[bb0445] Martin R.B., Burr D.B., Sharkey N.A., Martin R.B., Burr D.B., Sharkey N.A. (1998). Mechanical Properties of Ligament and Tendon, in Skeletal Tissue Mechanics.

[bb0450] Mashiatulla M., Ross R.D., Sumner D.R. (2017). Validation of cortical bone mineral density distribution using micro-computed tomography. Bone.

[bb0455] Maurer M.M. (2015). Does mechanical stimulation really protect the architecture of trabecular bone? A simulation study. Biomech. Model. Mechanobiol..

[bb0460] Milz S. (2002). Three-dimensional reconstructions of the Achilles tendon insertion in man. J. Anat..

[bb0465] Miserez A. (2008). The transition from stiff to compliant materials in squid beaks. Science.

[bb0470] Moffat K.L. (2008). Characterization of the structure-function relationship at the ligament-to-bone interface. Proc. Natl. Acad. Sci. U. S. A..

[bb0475] Nourissat G. (2010). Mesenchymal stem cell therapy regenerates the native bone-tendon junction after surgical repair in a degenerative rat model. PLoS One.

[bb0480] Nuzzo S. (2002). Quantification of the degree of mineralization of bone in three dimensions using synchrotron radiation microtomography. Med. Phys..

[bb0485] Palastanga N., Field D., Soames R. (2006). Anatomy and Human Movement: Structure and Function.

[bb0490] Paris O. (2000). Analysis of the hierarchical structure of biological tissues by scanning X-ray scattering using a micro-beam. Cellular and molecular biology (Noisy-le-Grand, France).

[bb0495] Paschalis E.P., Mendelsohn R., Boskey A.L. (2011). Infrared assessment of bone quality: a review. Clinical Orthopaedics and Related Research®.

[bb0500] Patel S. (2018). Integrating soft and hard tissues via interface tissue engineering. J. Orthop. Res..

[bb0505] Qu D. (2017). Compositional mapping of the mature anterior cruciate ligament-to-bone insertion. J. Orthop. Res..

[bb0510] Quental C. (2016). Full-thickness tears of the supraspinatus tendon: a three-dimensional finite element analysis. J. Biomech..

[bb0515] Razi H. (2015). Aging leads to a dysregulation in mechanically driven bone formation and resorption. J. Bone Miner. Res..

[bb0520] Razi H. (2020). Damage tolerance of lamellar bone. Bone.

[bb0525] Reznikov N. (2015). The 3D structure of the collagen fibril network in human trabecular bone: relation to trabecular organization. Bone.

[bb0530] Richardson W.J. (2018). Potential strain-dependent mechanisms defining matrix alignment in healing tendons. Biomech. Model. Mechanobiol..

[bb0535] Roschger P. (2008). Bone mineralization density distribution in health and disease. Bone.

[bb0540] Rossetti L. (2017). The microstructure and micromechanics of the tendon-bone insertion. Nat. Mater..

[bb0545] Rossi L.A. (2019). Current concepts in rotator cuff repair techniques: biomechanical, functional, and structural outcomes. Orthopaedic journal of sports medicine.

[bb0550] Rufai A., Ralphs J.R., Benjamin M. (1996). Ultrastructure of fibrocartilages at the insertion of the rat Achilles tendon. Journal of anatomy.

[bb0555] Ruffoni D., van Lenthe G.H., Ducheyne P. (2017). 3.10 Finite Element Analysis in Bone Research: A Computational Method Relating Structure to Mechanical Function☆, in Comprehensive Biomaterials II.

[bb0560] Ruffoni D. (2007). The bone mineralization density distribution as a fingerprint of the mineralization process. Bone.

[bb0565] Ruffoni D. (2008). Effect of temporal changes in bone turnover on the bone mineralization density distribution: a computer simulation study. J. Bone Miner. Res..

[bb0570] Ruffoni D., Müller R., van Lenthe G.H. (2012). Mechanisms of reduced implant stability in osteoporotic bone. Biomech. Model. Mechanobiol..

[bb0575] Saadat F. (2015). Effective elastic properties of a composite containing multiple types of anisotropic ellipsoidal inclusions, with application to the attachment of tendon to bone. Journal of the Mechanics and Physics of Solids.

[bb0580] Saadat F. (2016). The concentration of stress at the rotator cuff tendon-to-bone attachment site is conserved across species. J. Mech. Behav. Biomed. Mater..

[bb0585] Sano H., Saijo Y., Kokubun S. (2006). Non-mineralized fibrocartilage shows the lowest elastic modulus in the rabbit supraspinatus tendon insertion: measurement with scanning acoustic microscopy. J. Shoulder Elb. Surg..

[bb0590] Sano H., Wakabayashi I., Itoi E. (2006). Stress distribution in the supraspinatus tendon with partial-thickness tears: an analysis using two-dimensional finite element model. J. Shoulder Elb. Surg..

[bb0595] Sartori J., Stark H. (2020). Tracking tendon fibers to their insertion – a 3D analysis of the Achilles tendon enthesis in mice. Acta Biomater..

[bb0600] Sartori J. (2018). Three-dimensional imaging of the fibrous microstructure of Achilles tendon entheses in Mus musculus. J. Anat..

[bb0605] Schulte F.A. (2013). Local mechanical stimuli regulate bone formation and resorption in mice at the tissue level. PLoS One.

[bb0610] Schwartz A., Thomopoulos S., Thomopoulos S., Birman V., Genin G.M. (2013). The role of mechanobiology in the attachment of tendon to bone. Structural Interfaces and Attachments in Biology.

[bb0615] Schwartz A.G. (2012). Mineral distributions at the developing tendon enthesis. PLoS One.

[bb0620] Schwartz A.G., Long F., Thomopoulos S. (2015). Enthesis fibrocartilage cells originate from a population of hedgehog-responsive cells modulated by the loading environment. Development.

[bb0625] Sevick J.L. (2018). Fibril deformation under load of the rabbit Achilles tendon and medial collateral ligament femoral entheses. J. Orthop. Res..

[bb0630] Shaw H.M., Benjamin M. (2007). Structure–function relationships of entheses in relation to mechanical load and exercise. Scand. J. Med. Sci. Sports.

[bb0635] Shea J.E., Hallows R.K., Bloebaum R.D. (2002). Experimental confirmation of the sheep model for studying the role of calcified fibrocartilage in hip fractures and tendon attachments. Anat. Rec..

[bb0640] Simon D. (2015). The relationship between anterior cruciate ligament injury and osteoarthritis of the knee. Advances in orthopedics.

[bb0645] Sinclair K.D. (2011). Characterization of the anchoring morphology and mineral content of the anterior cruciate and medial collateral ligaments of the knee. Anat. Rec..

[bb0650] Spalazzi J.P. (2013). Quantitative mapping of matrix content and distribution across the ligament-to-bone insertion. PLoS One.

[bb0655] Spitzer V. (1996). The visible human male: a technical report. J. Am. Med. Inform. Assoc..

[bb0660] Teunis T. (2014). A systematic review and pooled analysis of the prevalence of rotator cuff disease with increasing age. J. Shoulder Elb. Surg..

[bb0665] Thomopoulos S. (2003). Variation of biomechanical, structural, and compositional properties along the tendon to bone insertion site. J. Orthop. Res..

[bb0670] Thomopoulos S. (2006). Collagen fiber orientation at the tendon to bone insertion and its influence on stress concentrations. J. Biomech..

[bb0675] Thomopoulos S., Birman V., Genin G.M. (2013). Structural Interfaces and Attachments in Biology.

[bb0680] Thunes J. (2015). The effect of size and location of tears in the supraspinatus tendon on potential tear propagation. J. Biomech. Eng..

[bb0685] Tuoheti Y. (2005). Contact area, contact pressure, and pressure patterns of the tendon-bone interface after rotator cuff repair. Am. J. Sports Med..

[bb0690] Wagermaier W. (2006). Spiral twisting of fiber orientation inside bone lamellae. Biointerphases.

[bb0695] Wagermaier W., Klaushofer K., Fratzl P. (2015). Fragility of bone material controlled by internal interfaces. Calcif. Tissue Int..

[bb0700] Waggett A.D. (1998). Characterization of collagens and proteoglycans at the insertion of the human achilles tendon. Matrix Biol..

[bb0705] Wakabayashi I. (2003). Mechanical environment of the supraspinatus tendon: a two-dimensional finite element model analysis. J. Shoulder Elb. Surg..

[bb0710] Wang Y., Ural A. (2018). Mineralized collagen fibril network spatial arrangement influences cortical bone fracture behavior. J. Biomech..

[bb0715] Wang H. (2014). Long-range force transmission in fibrous matrices enabled by tension-driven alignment of fibers. Biophys. J..

[bb0720] Wang J. (2015). Trabecular plates and rods determine elastic modulus and yield strength of human trabecular bone. Bone.

[bb0725] Weinkamer R., Fratzl P. (2016). Solving conflicting functional requirements by hierarchical structuring—examples from biological materials. MRS Bull..

[bb0730] Weinkamer R., Kollmannsberger P., Fratzl P. (2019). Towards a connectomic description of the osteocyte Lacunocanalicular network in bone. Current Osteoporosis Reports.

[bb0735] Wopenka B. (2008). The tendon-to-bone transition of the rotator cuff: a preliminary Raman spectroscopic study documenting the gradual mineralization across the insertion in rat tissue samples. Appl. Spectrosc..

[bb0740] Yamaguchi K. (2006). The demographic and morphological features of rotator cuff disease: a comparison of asymptomatic and symptomatic shoulders. JBJS.

[bb0745] Yao Q., Qu J. (2002). Interfacial versus cohesive failure on polymer-metal interfaces in electronic packaging—effects of interface roughness. J. Electron. Packag..

[bb0750] Zelzer E. (2014). Tendon-to-bone attachment: from development to maturity. Birth Defects Research Part C: Embryo Today: Reviews.

[bb0755] Zhang L. (2012). A coupled fiber-matrix model demonstrates highly inhomogeneous microstructural interactions in soft tissues under tensile load. J. Biomech. Eng..

[bb0760] Zickler G.A. (2012). Finite element modeling of the cyclic wetting mechanism in the active part of wheat awns. Biointerphases.

[bb0765] Zizak I. (2003). Characteristics of mineral particles in the human bone/cartilage interface. J. Struct. Biol..

[bb0770] Zorzetto L., Ruffoni D. (2019). Wood-inspired 3D-printed helical composites with tunable and enhanced mechanical performance. Adv. Funct. Mater..

